# Neurymenolide A, a Novel Mitotic Spindle Poison from the New Caledonian Rhodophyta *Phacelocarpus neurymenioides*

**DOI:** 10.3390/md17020093

**Published:** 2019-02-01

**Authors:** Sofia-Eléna Motuhi, Omid Feizbakhsh, Béatrice Foll-Josselin, Blandine Baratte, Claire Delehouzé, Arnaud Cousseau, Xavier Fant, Jeannette Chloë Bulinski, Claude Elisabeth Payri, Sandrine Ruchaud, Mohamed Mehiri, Stéphane Bach

**Affiliations:** 1 UMR ENTROPIE (IRD—Université de La Réunion—CNRS), Laboratoire d’Excellence Labex-CORAIL, Institut de Recherche pour le Développement (IRD), BP A5, 98848 Nouméa CEDEX, New Caledonia, France; sofia-elena.motuhi@sb-roscoff.fr (S.-E.M.); claude.payri@ird.fr (C.E.P.); 2 Sorbonne Université, CNRS, USR 3151, Protein Phosphorylation & Human Diseases, Station Biologique de Roscoff, CS 90074, 29688 Roscoff CEDEX, France; omid.feizbakhsh@sb-roscoff.fr (O.F.); beatrice.josselin@sb-roscoff.fr (B.F.-J.); baratte@sb-roscoff.fr (B.B.); claire.delehouze@gmail.com (C.D.); arnaud.cousseau@unice.fr (A.C.); xavierfant@yahoo.fr (X.F.); jcb4@columbia.edu (J.C.B.); sandrine.ruchaud@sb-roscoff.fr (S.R.); 3 UMR 7272 CNRS, Marine Natural Products Team, Nice Institute of Chemistry (ICN), University Nice Sophia Antipolis, Parc Valrose, 02 F-06108 Nice CEDEX, France; 4 Sorbonne Université, CNRS, UMR 8227, Integrative Biology of Marine Models, Station Biologique de Roscoff, CS 90074, 29688 Roscoff CEDEX, France; 5 Department of Biological Sciences, Columbia University, New York, NY 10027, USA

**Keywords:** New Caledonia, *Phacelocarpus neurymenioides*, Rhodophyta, neurymenolide A, mitotic spindle poison, mitotic catastrophe, necrosis, apoptosis

## Abstract

The marine α-pyrone macrolide neurymenolide A was previously isolated from the Fijian red macroalga, *Neurymenia fraxinifolia*, and characterized as an antibacterial agent against antibiotic-resistant strains that also exhibited moderate cytotoxicity in vitro against cancer cell lines. This compound was also shown to exhibit allelopathic effects on Scleractinian corals. However, to date no mechanism of action has been described in the literature. The present study showed, for the first time, the isolation of neurymenolide A from the New Caledonian Rhodophyta, *Phacelocarpus neurymenioides*. We confirmed the compound’s moderate cytotoxicity in vitro against several human cell lines, including solid and hematological malignancies. Furthermore, we combined fluorescence microscopy and flow cytometry to demonstrate that treatment of U-2 OS osteosarcoma human cells with neurymenolide A could block cell division in prometaphase by inhibiting the correct formation of the mitotic spindle, which induced a mitotic catastrophe that led to necrosis and apoptosis. Absolute configuration of the stereogenic center C-17 of neurymenolide A was deduced by comparison of the experimental and theoretical circular dichroism spectra. Since the total synthesis of this compound has already been described, our findings open new avenues in cancer treatment for this class of marine molecules, including a new source for the natural product.

## 1. Introduction

Cell division and programmed cell death constitute fundamental processes in the maintenance of tissue homeostasis. Indeed, dysfunctions in these mechanisms can lead to genetic instability, including numerical and structural chromosomal aberrations; the latter represents one of the first recognized attributes of cancer cells [1].

As reported in McGee’s review, the mitotic catastrophe was validated in 2012 by the International Nomenclature Committee on Cell Death as an “oncosuppressive mechanism that precedes and uses antiproliferative measures including apoptosis, necrosis, and senescence to prevent the proliferation of defective mitotic cells” [2,3]. Thus, mitotic catastrophe can arise from a variety of cellular damages and failures at cell-cycle checkpoints, in particular the spindle assembly checkpoint. This latter is used by cells to control the stable kinetochore-microtubule attachments of chromosomes during mitosis [3].

Microtubules are key cytoskeletal elements that take part in many different functions, notably in cell division. They are directly involved in the formation of the mitotic spindle, which is essential for segregating duplicated chromosomes into two identical sets, leading to two daughter cells at the end of mitosis [4]. Therefore microtubule-targeting drugs, usually known as “anti-mitotic”, represent important agents in anticancer chemotherapy. Their effectiveness has been proven by numerous clinical trials, including the remarkable success of taxanes (e.g., paclitaxel, Taxol^®^, Bristol-Myers Squibb, New York, NY, USA) and vinca alkaloids (e.g., vinblastine, Velbe^®^, Eurogenerics, Bruxelles, Belgium) which are mitotic spindle poisons commonly used to treat a wide variety of tumors, breast cancer, lung cancer, neuroblastoma, rhabdomyosarcoma, acute leukemia, Hodgkin’s disease, and non-Hodgkin’s lymphoma [5]. More specifically, vinca alkaloids work by inhibiting polymerization of tubulin into microtubules, while taxanes inhibit the depolymerization of microtubule into tubulin [6,7,8]. The therapeutic success of these agents foreshadowed the discovery of a number of novel microtubule-targeting drugs, such as eribulin mesylate (Halaven^®^, Eisai, Tokyo, Japan) and brentuximab vedotin (Adcetris^®^, Takeda, Osaka, Japan), two anti-mitotic agents derived from marine organisms that became available in 2010–2011. These drugs are used for patients with metastatic breast cancer who had previously received an anthracycline and a taxane for the treatment of relapsed or refractory Hodgkin’s lymphoma and systemic anaplastic large-cell lymphoma, respectively [9]. Eribulin mesylate [10,11] is a non-taxane microtubule dynamics inhibitor.

The second drug, brentuximab vedotin, is composed of an anti-CD30 chimeric antibody, an antibody that targets the cell membrane, coupled to monomethyl auristatin E, a derivative of dolastatin 10. Dolastatin 10 was first isolated by Pettit and co-workers from the Indian Ocean sea hare *Dolabella auricularia* [12] and was found to originate from the sea hare’s diet of marine cyanobacterium *Symploca* sp. [13]. The coupling of monomethyl auristatin E to anti-CD30 led to a highly effective and well-tolerated agent. Recently, several other novel marine natural products have begun preclinical and clinical trials, highlighting marine organisms as a promising source of anti-mitotic agents [9].

Neurymenolide A is a marine natural product that was first isolated from the Fijian Rhodophyta *Neurymenia fraxinifolia* (Mertens ex Turner) J. Agardh. Biological analyses showed moderately potent antibacterial activity against methicillin-resistant *Staphylococcus aureus* and vancomycin-resistant *Enterococcus faecium* with half-maximal inhibitory concentration (IC_50_) values of 2.1 µM and 4.5 µM, respectively. Further studies demonstrated moderate in vitro cytotoxicity against DU4475 breast tumor cells with IC_50_ of 3.9 µM, as well as mild to moderate activity against 11 other tumor cell lines with IC_50_ ranging from 5.4 to 28 µM [14]. A few years later, neurymenolide A was isolated from the Fijian Rhodophyta *Phacelocarpus neurymenioides* A.D.R. N’Yeurt, D.W. Keats and R.E. Norris as a coral bleaching-inducer on natural colonies of *Porites rus* [15]. The total synthesis of this compound was reported [16,17]. To date, no research has been carried out on the mechanism(s) of action of neurymenolide A, especially as regards its cytotoxic activity.

In keeping with the past 40 years of exploration of the New Caledonian marine chemodiversity (reviewed in Motuhi et al., 2016, Reference [18]), we have isolated and characterized neurymenolide A from the red macroalga *Phacelocarpus neurymenioides*. Along with a confirmation of the moderate effects of neurymenolide A on the viability of a set of human tumor cell lines, the current study helps to elucidate the cellular pathway affected by this marine natural product. Indeed, the results presented here suggest that neurymenolide A acts by destabilizing the mitotic spindle at the prometaphase transition in U-2 OS human osteosarcoma cells, which then results in mitotic catastrophe leading to necrosis and apoptosis. Moreover, our determination of the absolute configuration of the stereogenic carbon provides a helpful and necessary step toward elucidating the structure-activity relationship between neurymenolide A and its pharmacological action, particularly once its molecular targets have been identified precisely.

## 2. Results

### 2.1. Neurymenolide A Has Mild to Moderate Impact on the Growth of Cancerous Human Cell Lines

Neurymenolide A was screened for its effect on cell viability against a set of human cell lines selected to represent various cancer types: MCF-7 breast adenocarcinoma, HT-29 colorectal adenocarcinoma, Hep G2 hepatocellular carcinoma, IMR-32 neuroblastoma, U-2 OS osteosarcoma, AsPC-1 pancreatic adenocarcinoma, PANC-1 pancreatic epithelioid carcinoma, THP-1 acute monocytic leukemia, A3 acute T cell leukemia, K-562 chronic myelogenous leukemia, adriamycin-resistant K-562/ADR chronic myelogenous leukemia (Adriamycin^®^, Pfizer, New York, NY, USA) and HEK-293 embryonic kidney non-cancerous cell line.

The effect of neurymenolide A on cell viability was evaluated by a 3-(4,5-dimethylthiazol-2-yl)-5-(3-carboxymethoxyphenyl)-2-(4-sulfophenyl)-2*H*-tetrazolium (MTS) reduction assay.

Neurymenolide A decreased the viability of cancer cell lines in a 24-h assay, with half-maximal inhibitory concentrations (IC_50_) ranging from 73.3 to 446.5 µM (Table 1). The efficacy of cancer chemotherapy is critically dependent upon tumor cell selectivity. For example, a selectivity index (SI) value less than two indicates inadequate selective toxicity toward cancer cells [19]. As Table 1 shows, neurymenolide A is 3.3 times more potent towards A3 acute T cell leukemia than the normal human embryonic kidney cell line, HEK-293, in vitro. This compound also showed favorable selectivity toward THP-1, U-2 OS, and MCF-7 cell lines with SI values of 2.5, 2.4 and 2.3, respectively (Table 1).

U-2 OS, which was the adherent cell line most sensitive to neurymenolide A among those we assayed, was subsequently used to investigate the mechanism(s) by which this compound triggers cell death.

### 2.2. Neurymenolide A Induces Necrosis and Apoptosis in U-2 OS Human Osteosarcoma Cells

The apoptosis of osteosarcoma cells induced by neurymenolide A was demonstrated by flow cytometry analysis, performed following staining with Annexin V and Propidium-Iodide (PI).

**Figure 1:**
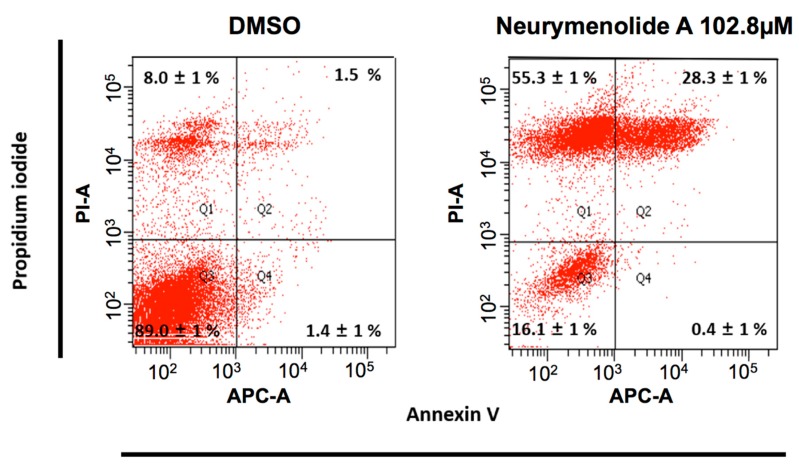
Characterization of apoptosis in human osteosarcoma cells by flow cytometry analysis after Annexin V and PI staining. PI^−^/Annexin V^−^, PI^−^/Annexin V^+^, PI^+^/Annexin V^+^ and PI^+^/Annexin V^−^ populations, corresponds to viable (Q3), early apoptotic (Q4), late apoptotic (Q2) and necrotic (Q1) cells, respectively. Values are expressed as means ± SD of two independent experiments.

Investigations revealed that 102.8 µM neurymenolide A induced a significant increase in the percentage of cells in both necrotic (55.3%) and late apoptotic (28.3%) cell subpopulations (Figure 1).

### 2.3. Neurymenolide A Induces Mitotic Spindle Destabilization at Prometaphase in U-2 OS Human Osteosarcoma Cells

**Figure 2:**
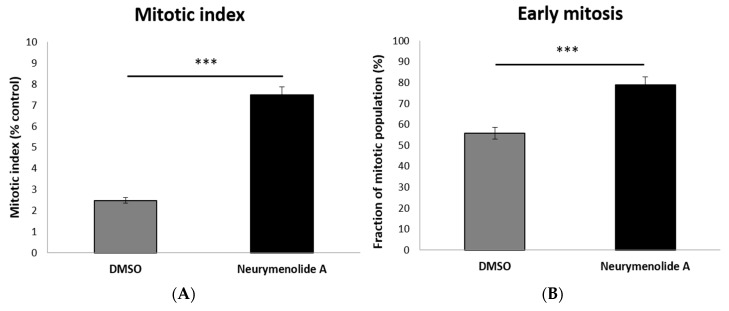
Effect of neurymenolide A treatment on the mitosis of osteosarcoma cells. (**A**) Assessment of the mitotic index, (**B**) assessment of the percentage of cells in the early stage of mitosis; both after exposure to neurymenolide A (102.8 µM) for 24 h. Histograms are representative of two independent experiments (n = 2, *** *p* < 0.001).

**Figure 3:**
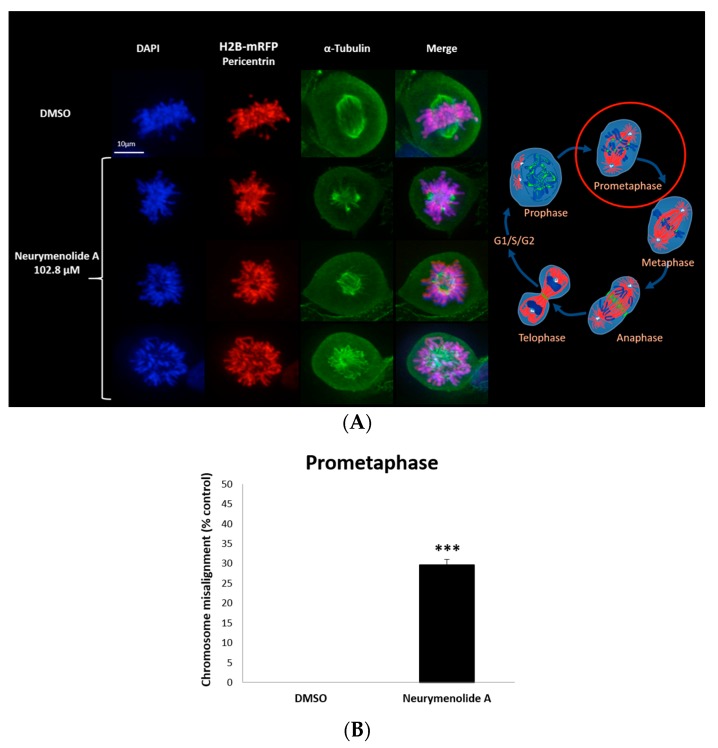
Effect of neurymenolide A treatment on the mitotic spindle of osteosarcoma cells. (**A**) Fluorescence micrographs showing morphology of U-2 OS human osteosarcoma cells incubated for 24 h with 102.8 µM neurymenolide A. U-2 OS cells stably expressing H2B-mRFP were stained for DAPI (blue), pericentrin (red) and α-tubulin (green). In merged images, red and green overlap appears yellow; blue and red overlap appears magenta. Scale bar = 10 µm. (**B**) Assessment of misalignment of chromosomes in prometaphase of early mitosis cells, following neurymenolide A incubation as in (**A**). Histograms are representative of two independent experiments (n = 2, *** *p* < 0.01).

As a first approach to study the mechanism(s) of action of neurymenolide A on cancer cell lines, we prepared time-lapse movies of living U-2 OS human osteosarcoma cells stably expressing H2B-mRFP (Histone2B-monomeric Red Fluorescent Protein). These time-lapse studies revealed that a 24 h treatment with 102.8 µM of neurymenolide A induced abnormal localization and dynamics of U-2 OS H2B-mRFP during cell division, whose genetic material looked irregularly star-shaped within the cell. To further explore cell division abnormalities, U-2 OS H2B-mRFP cells were fixed and stained for pericentrin (red), α-tubulin (green) and DAPI (blue) after a 24 h incubation period in the absence or presence of neurymenolide A. The mitotic index (MI), that is, the ratio of the number of cells in a population undergoing mitosis to the total number of cells, was also measured. These results are shown in Figure 2 and Figure 3.

MI values were 7.5% for treated cells and 2.5% for vehicle (DMSO), which suggests that the marine natural product induced a general decrease in the speed of mitosis, or perhaps arrested or slowed a specific phase of mitosis (Figure 2A). To distinguish these possibilities, we performed time-lapse to show that the number of cells in early mitosis dramatically increased over time (78.9%) after treatment with neurymenolide A, as cells entered mitosis, but failed to proceed through the later stages (Figure 2B). The images of the cells blocked in an aberrant, prophase-like stage are similar to C-mitosis, i.e., cells treated with colchicine (see for example Sirri et al., 2000, [20]).

Data gathered from image processing confirmed the star-shaped, C-mitosis-like chromosome distribution at the prometaphase transition (Figure 3A). Quantification revealed that 28.0% of cells in early mitosis showed a misalignment of chromosomes in prometaphase with disorganized spindles (*p* < 0.01) (Figure 3B); videomicroscopy demonstrated that these cells underwent mitotic arrest and apoptosis, including the formation of vesicles of cellular debris (see Supplementary Materials Section, Videos S1 and S2).

### 2.4. Neurymenolide A Induces a Delay of Microtubule Repolymerization in U-2 OS Human Osteosarcoma Cells

**Figure 4:**
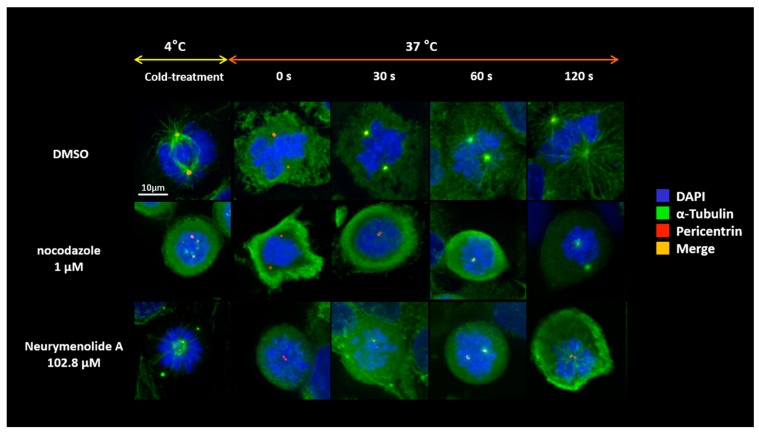
Microtubule repolymerization assay. Microtubule regrowth was monitored in U-2 OS cells in which microtubules had been cold-depolymerized (i.e., 1 h on ice). Repolymerization is shown at intervals of 0–120 s after shifting the temperature from 0° to 37 °C. Glass coverslips containing U-2 OS cells were fixed in methanol at −20 °C for 10 min, followed by immunofluorescence to visualize pericentrin (red) and α-tubulin (green), and staining with DAPI (blue), as described in the Experimental Section. In merged images, red and green combine to make yellow. Scale bar = 10 µm.

In order to gain mechanistic information on how neurymenolide A destabilizes the mitotic spindle, we pre-treated U-2 OS cells for 24 h in the absence or presence of our compound. We then performed an in cellulo microtubule repolymerization assay (Figure 4). Microtubules in pre-treated U-2 OS cells were depolymerized by cold treatment and then re-warmed to allow microtubules to repolymerize, still in the absence or presence of neurymenolide A. Nocodazole (Sigma-Aldrich, St. Louis, MO, USA), a known antagonist of microtubule polymerization, was used as a control.

The results shown in Figure 4 demonstrate both a delay in the re-polymerization of the microtubules in neurymenolide A-treated cells, compared to the DMSO control, and the inability to reorganize a spindle (Figure 4).

### 2.5. Neurymenolide A Has R Absolute Configuration at Position C-17

**Figure 5:**
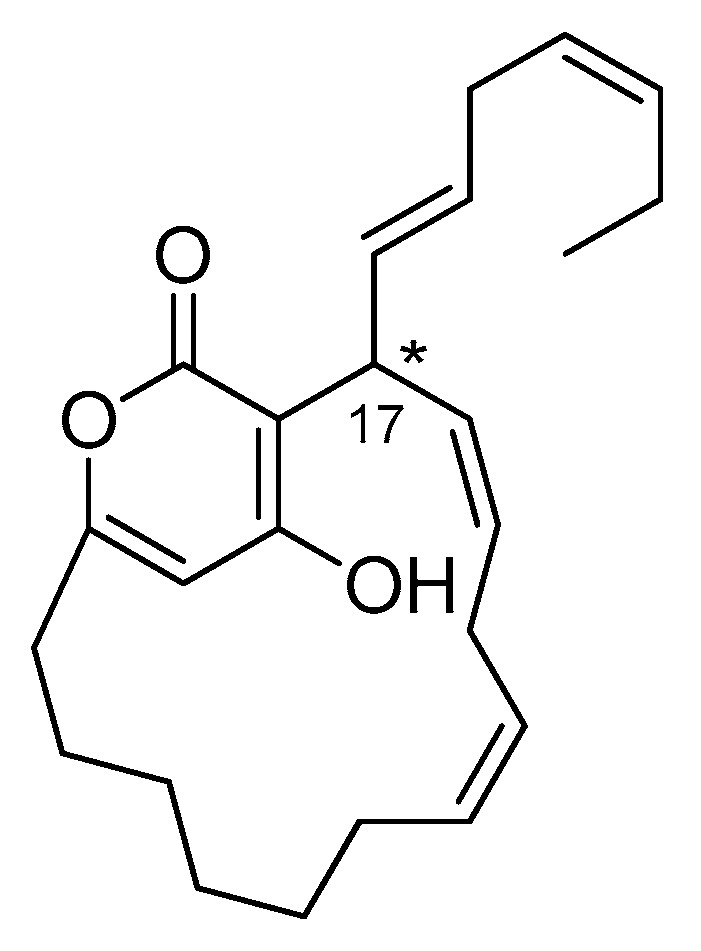
Structure of neurymenolide A.

Neurymenolide A is a polyunsaturated α-pyrone derivative isolated for the first time by Stout and his collaborators as two quickly interchanging atropisomers [14] (Figure 5, see also Supplementary Materials Section, Figure S1). Studies were carried out to determine the absolute configuration of its chiral center, including unsuccessful attempts to utilize crystal X-ray diffraction [16,17]. Despite significant efforts to date, the absolute configuration of C-17 of neurymenolide A had not been reported at the time of this study.

**Figure 6:**
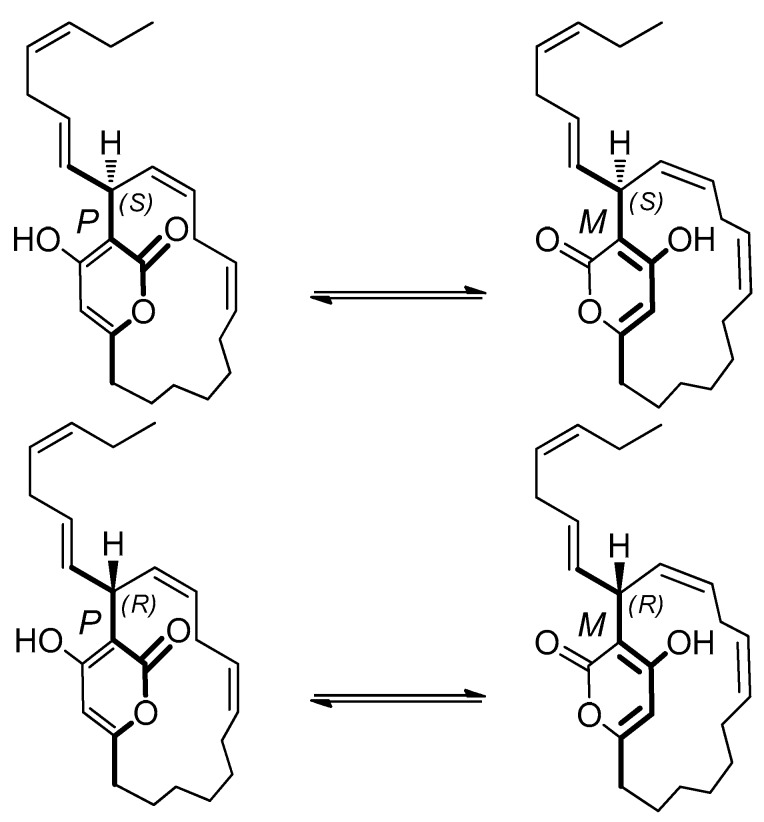
Structure of neurymenolide A configurations (*P*, *S*), (*M*, *S*), (*M*, *R*) and (*P*, *R*).

From a structural point of view, neurymenolide A shows a planar chirality due to the restricted rotation about the α-pyrone ring. Because this molecule contains a stereogenic center at C-17, the two atropisomers behave like diastereomers. Neurymenolide A configurations (*P*, *S*), (*M*, *S*), (*M*, *R*) and (*P*, *R*) are shown in Figure 6.

The chiral properties of neurymenolide A include several chromophores such as a pyrone and two double bonds (Figure 5). Notably, these chromophores are located near the stereogenic carbon C-17. Circular dichroism studies, which consist of comparing calculated ECD (electronic circular dichroism) spectra and experimental circular dichroism spectra, have been shown to be a powerful approach to determine the absolute configuration of chiral compounds that possess relevant chromophores near the stereogenic centers [21]. Accordingly, we exploited this method to determine the absolute configuration of the C-17 of neurymenolide A.

**Figure 7:**
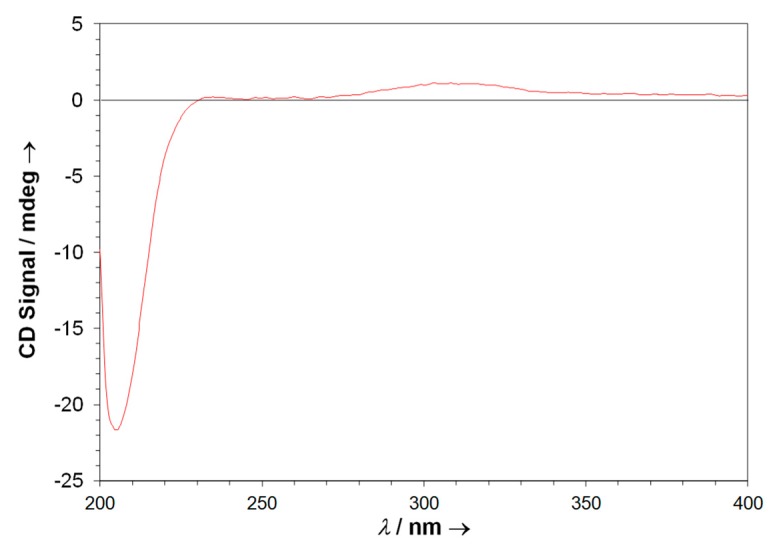
ECD experimental spectrum of neurymenolide A (1 µM, CH*3*OH).

An experimental CD spectrum of neurymenolide A revealed a weak positive Cotton effect at about 310 nm and a strong negative Cotton effect at about 205 nm (Figure 7).

**Figure 8:**
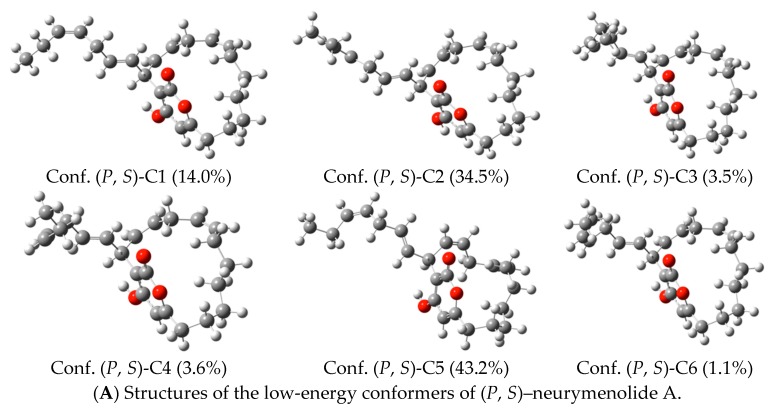

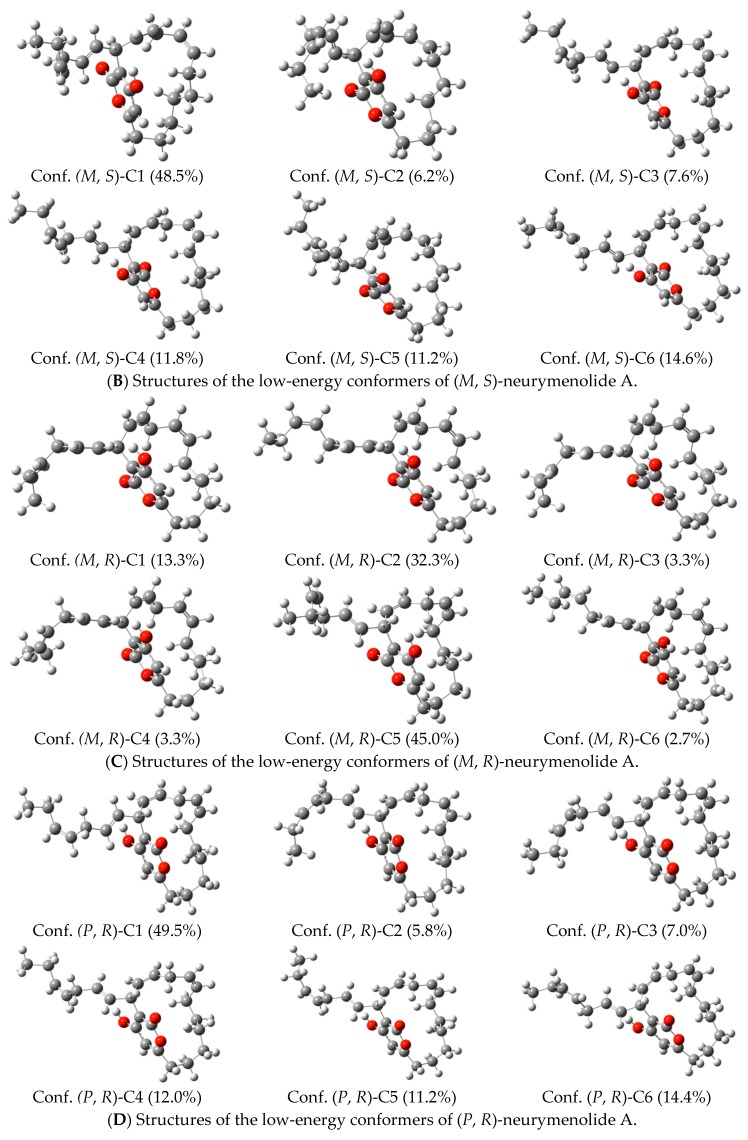
Structures and Boltzmann populations of the low-energy B3LYP/6-31+G(d,p) conformers (**A**–**D**) (*P*, *S*), (*M*, *S*), (*M*, *R*), and (*P*, *R*) configurations of neurymenolide A in CH_3_OH (298 K and 1 atm).

Next, theoretical ECD spectra were calculated based on neurymenolide A with (*P*, *S*), (*M*, *S*), (*M*, *R*) and (*P*, *R*) configurations (Figure 6), taking into account possible conformers. For each structure, the conformational analysis was performed at the semi-empirical level (AM1). These results are reported in the Supplementary Materials Section, Table S1. For each atropisomer, the resulting conformers of lower-energy were optimized using the density functional theory (DFT) method. All structures are presented in Figure 8 (see Supplementary Materials Section, Tables S2–S25, for more information).

**Figure 9:**
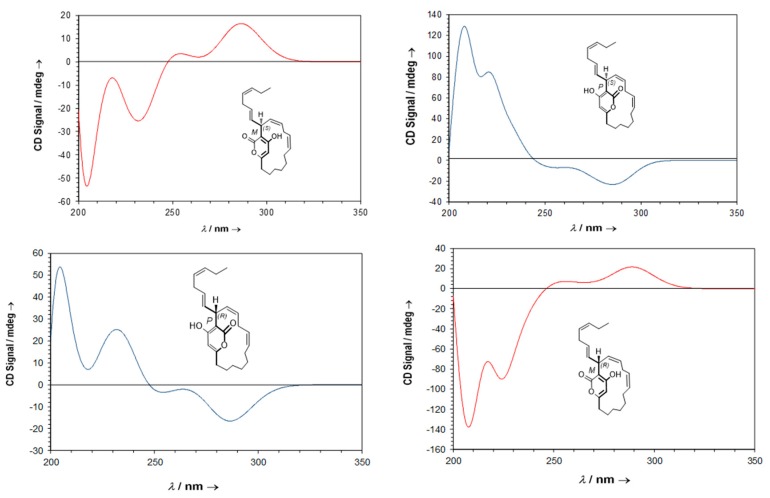
Theoretical ECD spectra of neurymenolide A of (*P*, *S*), (*M*, *S*), (*M*, *R*) and (*P*, *R*) configurations.

The ECD theoretical spectra were then calculated for each conformer by TDDFT (time-dependent density functional theory). The ECD total spectra, weighted-average Boltzmann associated to each conformer, were then simulated (Figure 9).

**Figure 10:**
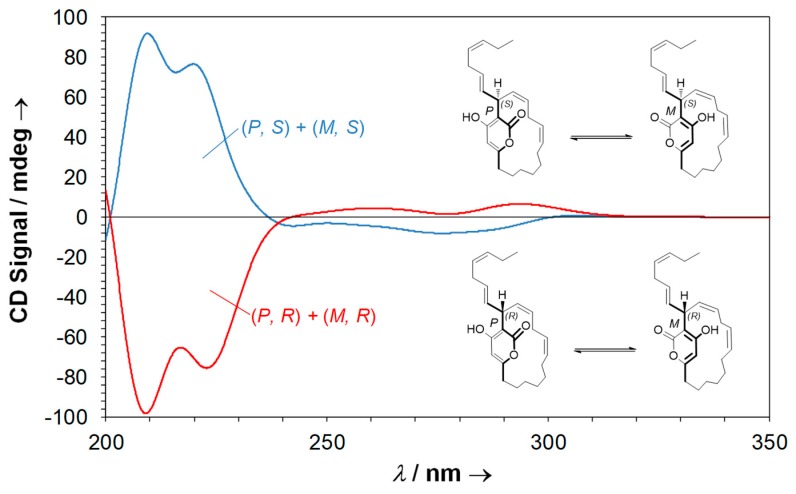
Theoretical ECD spectra of both possible equimolar mixtures of neurymenolide A.

These results allowed us to calculate the theoretical ECD spectra of the two equimolar mixtures of neurymenolide A that are possible, (*P*, *S*) + (*M*, *S*) and (*P*, *R*) + (*M*, *R*) (Figure 10).

Taken together, the experimental and theoretical ECD spectra (calculated at B3LYP/6-31+G(d,p) level) allowed us to assign the absolute configuration of neurymenolide A as 17*R*. 

## 3. Discussion

Although neurymenolide A was previously investigated for its antiproliferative activity, the previous experiments did not address its putative mechanism(s) of action. Accordingly, in this paper, we paid particular attention to the mechanism(s) by which this marine natural product modulated the viability of cancer-derived cell lines in vitro. After confirming the cell growth-inhibiting properties of hundred-micromolar levels of this compound against several human cancer cells, we determined that neurymenolide A could act as a novel mitotic spindle poison. Indeed, our work demonstrates that neurymenolide A significantly delays the in vivo polymerization of tubulin to form microtubules and bi-polar mitotic spindles at the prophase-metaphase transition. Moreover, our results are consistent with the notion that mitotic perturbations induced by neurymenolide A trigger mitotic catastrophe that then results in cell death. Future studies, particularly in vitro experiments, will be required in order to answer basic questions concerning its mechanism(s) of action in deranging the mitotic spindle apparatus. For example, does neurymenolide A bind directly to tubulin subunits, or, more likely, to components of the microtubule organizing center or mitotic motors such as KiF11 (Eg5)? Does it have other bioactivities that are deleterious to cells? Further work will be needed to address these questions.

Notably, in our experiments, neurymenolide A concentrations that prevented mitotic progression or killed cells were much greater (IC_50_ > 100 μM) than the concentrations commonly used for therapeutic drugs (generally 1–100 nM). This suggests a number of possibilities: the affinity of neurymenolide A for its target(s) is quite low; when bound to its target protein(s) its inhibition of the activity of targets is only partial; its hydrophobicity limits the amount of active neurymenolide A the cells are exposed to in aqueous culture medium; or (perhaps the most likely) the atropisomers differ in their potency and a racemic mixture therefore contains both highly active and less active forms of the drug. In addition, 102.8 μM and 46.5 μM are the IC_50_ observed for U-2 OS cells treated with neurymenolide A for 24 h and 72 h, respectively; a longer incubation with the drug yielded cell death/mitosis prevention at lower neurymenolide A concentrations. In agreement with our results, Stout et al., 2009 [14] treated cells with neurymenolide A for 72 h and reported the killing of cell lines with IC_50_ ranging from 3.9–28 μM. We utilized the shorter incubation time in our study as it optimized our ability to explore the mechanistic contributors to cell killing.

Drug-induced mitotic catastrophe represents a promising new approach in cancer prevention and treatment [3]. In addition to microtubules, a number of potential mitotic targets have been exploited, such as motor proteins or mitotic kinases [22,23]. Mitotic spindle kinases that are potential targets include monopolar spindle 1 (Mps1), polo-like kinases (e.g., Plk1) and aurora kinases (e.g., Aurora B) since all play critical roles during mitosis [24,25,26]. Thus far we have performed a molecular screen on a panel of kinase proteins that included *Hs*Aurora-B. However, neurymenolide A showed no inhibitory activity towards Aurora B (data not shown).

Neurymenolide A is an equimolar mixture of two atropisomers and, thus, there are two possible forms of the drug. More work will be required to define how quickly each atropisomer can racemize and under what conditions this occurs. If the stereochemistry of both atropisomer is Class III (very stable; see review by Glunz, 2018 for more information, [27]), then separation of each of the two atropisomers will be possible and we can test purified versions of each enantiomer to determine if each works with an identical mechanism and potency. On the other hand, if each racemizes with a very short half-time t_½,_ (Class I) or an intermediate t_½_ (minutes to months) we will try to derivatize the molecule so as to give it the properties of a Class III molecule, since we can then purify and separately analyze the activity of each of the two atropisomers. Potency differences may be profound and such studies may yield a form of neurymenolide A that is significantly more potent or target specific, as has been noted for protein kinase inhibitors [28].

Aside from the discovery that neurymenolide A induced mitotic arrest and cell death, this study also elucidated an absolute configuration, which was designated 17*R*. It is important to note that since its first isolation in 2009, the absolute configuration of neurymenolide A had not been established, in part because of the molecule’s unstable conformation [16,17]. Therefore, establishing the structure will aid in forthcoming structure-activity relationship studies, particularly in vitro studies of neurymenolide A and tubulin.

Taken together, the data described here reveal a new putative mitotic spindle poison, neurymenolide A, isolated for the first time from a New Caledonian species of macroalgae. This work has also opened interesting avenues with regards to the mechanism(s) by which this natural product may affect the mitotic spindle and potentially the polymerization of tubulin, a very suitable target for the development of cancer treatments. The key step will now be to lower the effective concentration of the molecule by designing new neurymenolide derivatives and/or by testing synergetic effects with other anticancer drugs [29], in order to render neurymenolide A amenable for use in clinical trials [30]. Neurymenolide A may thus be considered as a novel prototype that could eventually be useful in designing new anticancer drug therapies involving the induction of mitotic catastrophe.

## 4. Materials and Methods

### 4.1. Reagents

Phosphate buffered saline (PBS), trypsin/EDTA solution 0.05% and dimethylsulfoxide (DMSO) were purchased from Life Technologies^TM^ (Thermo Fisher Scientific, Waltham, MA, USA). Analytical grade organic solvents (methanol, dichloromethane) were purchased from Merck (Darmstadt, Germany). Water, acetonitrile, ethyl acetate and cyclohexane used for HPLC were of HPLC-grade and purchased from Sigma-Aldrich^®^, St. Louis, MO, USA. HPLC-grade formic acid was purchased from ACROS Organics, Thermo Fisher Scientific, Waltham, MA, USA.

### 4.2. Biological Material

The Rhodophyta *Phacelocarpus neurymenioides* (*Florideophyceae*, *Gigartinales*, *Phacelocarpaceae*) was collected from Nouméa, in the South lagoon at 70 m deep by SCUBA diving in October 2014. The specimen was identified by Dr. C.E. Payri (IRD Nouméa).

### 4.3. Extraction and Isolation of Neurymenolide A

The MeOH/CH_2_Cl_2_ (1:1, *v/v*) crude organic extract of *Phacelocarpus neurymenioides* (0.94 g) was obtained after an exhaustive extraction of 17.4 g of dry powder. After that, the crude extract was fractionated by Sephadex LH20 eluted with a mixture of MeOH/CH_2_Cl_2_ (1:1, *v/v*). Next, 36.0 mg of the subsequent fraction (240.5 mg) was purified by semi-preparative high-performance liquid chromatography (HPLC) in normal-phase (Hypersil Lichrosorb Diol, 250 × 10 mm id, 5 µm, gradient EtOAc/*n*-hexane, 30:70 to 100:0, flow rate: 3.0 mL/min) to afford pure neurymenolide A (26.1 mg) as an amorphous solid. 

The ^1^H NMR (500 MHz) and ^13^C NMR (125 MHz) (Table 2) data are in agreement with published data (see Supplementary Materials Section, Figures S2 and S3, for more information) [16].

#### Neurymenolide A

^1^H NMR (400 MHz, CDCl_3_, *δ*/ppm, *J*/Hz): 6.32 (1H, br s, OH), 5.80 (1H, s, H-4), 5.65 (2H, m, H-15, H-16), 5.61 (2H, m, H-18, H-19), 5.42 (1H, m, H-22), 5.30 (1H, m, H-21), 5.23 (1H, m, H-12), 5.21 (1H, m, H-13), 4.58 (1H, br s, H-17), 2.85 (1H, m, H-14), 2.71 (1H, m, H-17), 2.60 (1H, ddd, *J* = 14.0, *J* = 11.0, *J* = 3.3, H-6), 2.52 (1H, m, H-14), 2.30 (1H, ddd, *J* = 14.0, *J* = 11.0, *J* = 3.3, H-6), 2.00 (2H, p, *J* = 15.0, *J* = 7.4, H-23), 1.86 (1H, m, H-7), 1.83 (1H, m, H-11), 1.76 (1H, m, H-11), 1.57 (1H, m, H-7), 1.35 (1H, m, H-9), 1.34 (1H, m, H-8), 1.28 (1H, m, H-8), 1.18 (1H, m, H-9), 1.15 (1H, m, H-10), 1.07 (1H, m, H-10), 0.94 (3H, t, *J* = 7.4, H-24).

^13^C NMR (100 MHz, CDCl_3_, *δ*/ppm): 165.1 (C-1, C-5), 164.5 (C-3), 135.6 (C-15), 133.0 (C-22), 131.0 (C-12), 129.9 (C-19), 129.4 (C-18), 126.8 (C-16), 126.6 (C-13), 125.9 (C-21), 103.8 (C-2), 101.3 (C-4), 36.5 (C-17), 33.5 (C-6), 30.0 (C-20), 27.7 (C-9), 27.1 (C-10), 27.0 (C-14), 26.9 (C-8), 26.6 (C-11), 25.6 (C-7), 20.5 (C-23), 14.2 (C-24).

HR-ESI-MS: *m/z* 737.4792 [2M+H]^+^.

### 4.4. Cell Lines and Cell Culture Conditions

Cell lines were obtained from the American Type Culture Collection (ATTCC; Manassas, VA, USA) and comprised U-2 OS human osteosarcoma (ATCC^®^ HTB-96^TM^), HT-29 human colorectal adenocarcinoma (ATCC^®^ HTB-38^TM^), MCF7 human breast adenocarcinoma (ATCC^®^ HTB-22^TM^), PANC-1 human pancreas epithelioid carcinoma (ATCC^®^ CRL-1469^TM^), AsPC-1 human pancreas adenocarcinoma (ATCC^®^ CRL-1682^TM^), IMR-32 human brain neuroblastoma (ATCC^®^ CCL-127^TM^), Hep G2 human hepatocellular carcinoma (ATCC^®^ HB-8065^TM^), A3 acute T cell leukemia (ATCC-CRL-2570), K-562 WT human chronic myelogenous leukemia (ATCC^®^ CCL-243^TM^) and HEK-293 human embryonic kidney (ATCC^®^ CRL-1573^TM^). U-2 OS human osteosarcoma cells stably expressing H2B-mRFP were obtained using the protocol of Fant et al., 2009 [31]. The cells were maintained as monolayers at 37 °C and 5% CO_2._ The culture media, used interchangeably, were RPMI-1640 or DMEM Dulbecco’s modified eagle medium; the media were supplemented with 10 to 15% fetal bovine serum (Life Technologies^TM^, Thermo Fisher Scientific, Waltham, MA, USA).

### 4.5. Cell Treatments

Neurymenolide A was dissolved in DMSO and stored at −20 °C. The cells were treated for 24 or 72 h with neurymenolide A, vehicle (DMSO), doxorubicin (Adriamycin^®^, Pfizer, New York, NY, USA) or nocodazole.

### 4.6. MTS Reduction Assay

The effect of tested compounds on cell viability was assessed by MTS (3-(4,5-dimethylthiazol-2-yl)-5-(3-carboxymethoxyphenyl)-2-(4-sulfophenyl)-2H-tetrazolium) reduction assay. Cell suspension of 0.5 × 10^5^/mL (AsPC-1, MCF7, U-2 OS and PANC-1), 0.4 × 10^6^/mL (IMR32), 0.2 × 10^6^/mL (Hep G2, A3, THP-1, K562, K562/adr), 0.25 × 10^5^/mL (HT-29) and 0.1 × 10^6^/mL (HEK-293) were distributed in 96-well plates and incubated at 37 °C and 5% CO_2_ in the absence or presence of increasing concentrations of neurymenolide A from 0.1 to 676.6 µM for 24 or 72 h. After incubation with the compounds, 30 µL of MTS solution (CellTiter 96^®^ Aqueous One Solution Cell Proliferation Assay, Promega, Fitchburg, WI, USA) was added to each well and the plates were incubated for 3 h at 37 °C with 5% CO_2_. The plates were then read on BioTek Instruments EL800 Microplate Reader at 490–630 nm and the signals were analyzed using Gen5 software. The IC_50_ values (concentration of the drug at which cell growth was inhibited by 50%) were generated from the dose-response curves and were representative of at least four independent experiments performed in triplicate. Cell viability was expressed as a percentage, calculated as follows: Cell viability rate (%) = (A treatment/A control) × 100. 

### 4.7. Flow Cytometry Analysis

The induction of apoptosis by neurymenolide A was assessed by flow cytometry using the FlowCellect Annexin Red kit (Merck Millipore, Billerica, MA, USA) and PI/RNase staining buffer (BD Pharmingen^TM^, BD Biosciences, San Diego, CA, USA). U-2 OS cells (5 × 10^5^ cells/well) were distributed in 6-well plates and incubated in the absence or presence of neurymenolide A (102.8 µM) in a complete growth medium for 24 h. Cells were then washed and stained with Annexin V and propidium iodide, following the manufacturer’s instructions. Data acquisition and analysis were performed by a BD FACsAria^TM^ flow cytometer (BD Biosciences, San Jose, CA, USA) using BD FACSDiva^TM^ software v. 6.1.3 and the PI^−^/Annexin V^−^, PI^−^/Annexin V^+^, PI^+^/Annexin V^+^ and PI^+^/Annexin V^−^ populations, corresponding to viable (Q3), early apoptotic (Q4), late apoptotic (Q2) and necrotic (Q1) cells, respectively.

### 4.8. Immunofluorescence Staining

U-2 OS H2B-mRFP cells (5 × 10^5^ cells/Petri dishes) were incubated in the absence or presence of neurymenolide A (102.8 µM) for 24 h at 37 °C and 5% CO_2_. The cells were fixed with freshly prepared 4% paraformaldehyde for 10 min at room temperature (RT). Following three 5 min washes in PBS, the cells were mildly permeabilized with PBS containing 0.15% Triton for two minutes at RT. The cells were washed thrice in PBS for five minutes each and then blocked with 1% bovine serum albumin at RT for one hour. Incubation with primary antibodies was performed (1:1000 dilution of rabbit polyclonal pericentrin antibody, Novus NB.100-61071 1 mg/mL; 1:1000 dilution of mouse monoclonal α-tubulin antibody, Sigma-Aldrich T5168 100 U/mL), washed intensively and incubated with a 1:1000 dilution of the appropriate secondary antibodies labeled with Alexa 488 and Alexa 594 dyes (Invitrogen). After staining, coverslips were mounted in Mowiol with 4′,6-diamidino-2-phenylindole DAPI (Mounting Medium for fluorescence with DAPI, H1200, Vector Laboratoire, lot: ZA111). Data acquisition and image processing were performed by a Microscope Zeiss (camera cool SNAP HQ2, 100X objective) using MetaMorph^®^ (MetaMorph Inc., Nashville, TN, USA) and ImageJ (National Institutes of Health, Bethesda, MD, USA) software. 

### 4.9. Time-Lapse Movies

U-2 OS H2B-mRFP cells were treated at 37 °C and 5% CO_2_ in the absence or presence of neurymenolide A (102.8 µM) for eight hours. The cells were then observed under a fluorescence microscope for 16 h at 37 °C. Data acquisition, including image capture (one image every 15 min) and image processing were performed on a Zeiss Microscope as described above.

### 4.10. Microtubule Regrowth Assay

U-2 OS cells were incubated at 37 °C and 5% CO_2_ for 24 h in the absence or presence of neurymenolide A (102.8 µM). As a positive control, the cells were also treated with nocodazole (1 µM for 12 h), which is known to interfere with the polymerization of microtubules for 12 h; as a negative control cells were incubated with the vehicle alone (0.08% DMSO). Following drug treatment, coverslips were transferred into pre-cooled medium on ice for one hour. Coverslips were removed with forceps and immediately immersed into a pre-warmed medium at 37 °C for defined time intervals (0, 30, 60 and 120 s). Regrowth was then stopped by methanol fixation (10 min at −20 °C). Thereafter, incubation with primary pericentrin and α-tubulin antibodies was performed, coverslips were then washed intensively and incubated with appropriate secondary antibodies, and processed and imaged as described above in Section 4.8.

### 4.11. Circular Dichroism Data

Circular dichroism data were obtained using a J-810 spectropolarimeter (Jasco). TDDFT calculations of electronic circular dichroism (ECD) were performed with Gaussian 09 software (Gaussian Inc., Wallingford, CT, USA). The analysis of the distribution of the conformers and the optimized geometries were obtained using the semi empiric force field AM1 found in Spartan 08 software (Wavefunction Inc. Irvine, CA, USA). For each derivative, the structures were screened and filtered in order to avoid duplication. Then, each conformer was optimized in regards to the geometry using the DFT hybrid method B3LYP with the calculation bases 6-31+G(d,p) (B3LYP/6-31+G(d,p)), leading to thermochemical parameters at 298 K and 1 atm. The effects of solvation have been modeled using a continuous polarization model (PCM). Based on the TDDFT calculations carried out on the structure of each optimized conformer, the excitation energies (in nm) and the rotational force, R, broken down into velocity dipole (R_vel_) and length dipole (R_len_), were simulated using an ECD curve with the subsequent Gaussian function:

$$\Delta \varepsilon \left(E\right)=\;\overset{n}{\underset{i=1}{\sum }}\Delta \varepsilon_{i}\;\left(E\right)=\;\overset{n}{\underset{i=1}{\sum }}\left(\frac{RiEi}{2.29\times 10{\;}^{-39}\sqrt{\pi \sigma }}exp\left[-\;{\left(\frac{E-Ei}{\sigma }\right)}^{2}\right]\right)\;$$

where *σ* is the width of the band at 1/e peak-height, *Ei* and *Ri* are the excitation energy and rotator strength for transition *i*, respectively. Values of *σ* = 0.20 eV and R_vel_ were used. SpecDis 1.60 software was employed to generate the spectra in Gaussian shape. The ECD spectra Boltzmann populations-weighted were obtained from the structures which were optimized at B3LYP/6-31+G(d,p) level.

### 4.12. Statistical Analysis

All data are expressed as mean ± standard deviation (SD). A Chi-squared test was used to compare the actual difference between the two populations. *p* < 0.01 was considered to be statistically significant and *p* < 0.001 highly significant.

## 5. Conclusions

In summary, the data here show that the α-pyrone macrolide neurymenolide A, isolated from the New Caledonian Rhodophyta *P. neurymenioides*, is able to induce a mitotic catastrophe, as well as cell death by both necrosis and apoptosis in U-2 OS human osteosarcoma cells.

To the best of our knowledge, our work constitutes a first attempt to address the mechanism of action by which neurymenolide A inhibits the division of cancer cells. Our results suggest that this marine natural product may act on spindle organization by delaying microtubule polymerization during the prometaphase transition. Further studies are underway to definitively identify neurymenolide A’s targets and to explore structure-activity relationships.

In summary, our findings could possibly lead to strategies to exploit mitotic catastrophe for the treatment of cancer, especially for malignancies that are currently not amenable to treatment.

## Figures and Tables

**Table 1:** Effect of a treatment by neurymenolide A on the viability (IC_50_) of various cell lines.

Cancer Type	Cell Line	IC_50_ (µM)	SI **
Solid malignancies	U-2 OS	102.8 ± 1 (46.5) *	2.4
	MCF-7	107.4	2.3
	IMR-32	181.9 ± 1	1.3
	Hep G2	192.1	1.3
	PANC-1	270.6 ± 2	0.9
	HT-29	362.1	0.7
	AsPC-1	446.5 ± 1	0.6
Hematological malignancies	A3	73.3 (27.9) *	3.3
	THP-1	99.5	2.5
	K-562/ADR	243.6 ± 1	1.0
	K-562	398.6 ± 1	0.6
Non-malignant	HEK-293	243.6	-

Cells (5.0 × 10⁴ to 4.0 × 10⁵/mL) were incubated for 24 h at 37 °C and 5% CO_2_ in the presence of increasing doses of neurymenolide A (from 0.1 to 676.6 µM). Cell viability was measured by MTS reduction assay as indicated in the Materials and Methods section. IC_50_ values were calculated from the dose-response curves and are representative of at least four independent experiments made in triplicate. * The effect of the treatment was also tested at 72 h for U-2 OS and A3. The values obtained are shown in brackets. ** Selectivity index (SI): IC_50_ of pure compound on normal cell line divided by the IC_50_ of pure compound on cancer cell line.

**Table 2:** Comparison of the ^13^C NMR data of isolated neurymenolide A with published data (natural products and synthetic samples).

	Natural Products			Synthetic
N°	*δ*_c_ (ppm) ^a^	*δ*_c_ (ppm) ^b^	Δ*δ_c_*	*δ*_c_ (ppm) ^c^
1	165.1	165.1	0	165.1
2	103.8	103.9	0.1	103.8
3	164.5	164.7	0.2	164.7
4	101.3	101.4	0.1	101.3
5	165.1	165.1	0	165.1
6	33.5	33.5	0	33.5
7	25.6	25.6	0	25.6
8	26.9	26.9	0	26.9
9	27.7	27.7	0	27.7
10	27.1	27.1	0	27.1
11	26.6	26.6	0	26.6
12	131.0	131.0	0	131.0
13	126.6	126.6	0	126.6
14	27.0	27.0	0	27.0
15	135.6	135.2	−0.4	135.5
16	126.8	127.0	0.2	126.9
17	36.5	36.5	0	36.5
18	129.4	129.4	0	129.4
19	129.9	129.9	0	129.9
20	30.0	30.0	0	30.0
21	125.9	125.9	0	125.9
22	133.0	132.9	−0.1	133.0
23	20.5	20.5	0	20.5
24	14.2	14.2	0	14.2

^a^ Neurymenolide A isolated from *Phacelocarpus neurymenioides*, CDCl_3_, 100 MHz; ^b^ natural product sample of neurymenolide A isolated from *Neurymenia fraxinifolia*, CDCl_3_, 150 MHz [14]; ^c^ synthetic sample of neurymenolide A [16], CDCl_3_, 125 MHz.

## Supplementary Materials

The following are available online at http://www.mdpi.com/1660-3397/17/2/93/s1, Video S1: Time-lapse imaging in living human osteosarcoma cells treated with control (DMSO), Video S2: Time-lapse imaging in living human osteosarcoma cells treated with neurymenolide A, Figure S1: HPLC-UV-ELSD chromatogram of two equimolar mixtures of neurymenolide A, Figure S2: 1H NMR spectrum of neurymenolide A in CDCl3 at 400 MHz, Figure S3: 13C NMR spectrum of neurymenolide A in CDCl3 at 100 MHz, Table S1: Etotal, Boltzmann factor, ΔE and % for atropisomers of neurymenolide A, Table S2: Coordinates (Ångstroms) for conformer (*P*, *S*)-C1 of neurymenolide A, Table S3: Coordinates (Ångstroms) for conformer (*P*, *S*)-C2 of neurymenolide A, Table S4: Coordinates (Ångstroms) for conformer (*P*, *S*)-C3 of neurymenolide A, Table S5: Coordinates (Ångstroms) for conformer (*P*, *S*)-C4 of neurymenolide A, Table S6: Coordinates (Ångstroms) for conformer (*P*, *S*)-C5 of neurymenolide A, Table S7: Coordinates (Ångstroms) for conformer (*P*, *S*)-C6 of neurymenolide A, Table S8: Coordinates (Ångstroms) for conformer (*M*, *S*)-C1 of neurymenolide A, Table S9: Coordinates (Ångstroms) for conformer (*M*, *S*)-C2 of neurymenolide A, Table S10: Coordinates (Ångstroms) for conformer (*M*, *S*)-C3 of neurymenolide A, Table S11: Coordinates (Ångstroms) for conformer (*M*, *S*)-C4 of neurymenolide A, Table S12: Coordinates (Ångstroms) for conformer (*M*, *S*)-C5 of neurymenolide A, Table S13: Coordinates (Ångstroms) for conformer (*M*, *S*)-C6 of neurymenolide A, Table S14: Coordinates (Ångstroms) for conformer (*M*, *R*)-C1 of neurymenolide A, Table S15: Coordinates (Ångstroms) for conformer (*M*, *R*)-C2 of neurymenolide A, Table S16: Coordinates (Ångstroms) for conformer (*M*, *R*)-C3 of neurymenolide A, Table S17: Coordinates (Ångstroms) for conformer (*M*, *R*)-C4 of neurymenolide A, Table S18: Coordinates (Ångstroms) for conformer (*M*, *R*)-C5 of neurymenolide A, Table S19: Coordinates (Ångstroms) for conformer (*M*, *R*)-C6 of neurymenolide A, Table S20: Coordinates (Ångstroms) for conformer (*P*, *R*)-C1 of neurymenolide A, Table S21: Coordinates (Ångstroms) for conformer (*P*, *R*)-C2 of neurymenolide A, Table S22: Coordinates (Ångstroms) for conformer (*P*, *R*)-C3 of neurymenolide A, Table S23: Coordinates (Ångstroms) for conformer (*P*, *R*)-C4 of neurymenolide A, Table S24: Coordinates (Ångstroms) for conformer (*P*, *R*)-C5 of neurymenolide A, Table S25: Coordinates (Ångstroms) for conformer (*P*, *R*)-C6 of neurymenolide A.
