# Supplementary material for: Neurymenolide A, a Novel Mitotic Spindle Poison from the New Caledonian Rhodophyta Phacelocarpus neurymenioides
Source: Mar Drugs. 2019 Feb 1;17(2):93. doi: 10.3390/md17020093 (PMC6410418; doi:10.3390/md17020093)
Supplement: Supplementary file 1 [file marinedrugs-17-00093-s001.zip › marinedrugs-419240-SI-for final-V3/marinedrugs-419240-2_Supplementary_Proof_Final.pdf]

SUPPLEMENTARY MATERIALS

Neurymenolide A, a novel mitotic spindle poison from the New Caledonian Rhodophyta *Phacelocarpus neurymenioides*.

Sofia-Eléna Motuhi [1,2,3], Omid Feizbakhsh [2,4], Béatrice Foll-Josselin [2], Blandine Baratte [2], Claire Delehouzé [2], Arnaud Cousseau [2,3], Xavier Fant [2], Jeannette Chloë Bulinski [2,5], Claude Elisabeth Payri [1], Sandrine Ruchaud [2], Mohamed Mehiri [3,*] and Stéphane Bach [2,*]

[1] UMR ENTROPIE (IRD—Université de La Réunion—CNRS), Laboratoire d'Excellence Labex-CORAIL, Institut de Recherche pour le Développement (IRD), BP A5, Nouméa Cedex 98848, Nouvelle-Calédonie; sofia-elena.motuhi@sb-roscoff.fr (S.-E.M.); claude.payri@ird.fr (C.E.P.)

[2] Sorbonne Université, CNRS, USR 3151, Protein Phosphorylation & Human Diseases, Station Biologique de Roscoff, CS 90074, 29688 Roscoff Cedex, France; beatrice.josselin@sb-roscoff.fr (B.F.-J.); baratte@sb-roscoff.fr (B.B.); claire.delehouze@gmail.com (C.D.); xavierfant@yahoo.fr (X.F.); sandrine.ruchaud@sb-roscoff.fr (S.R.)

[3] UMR 7272 CNRS, Marine Natural Products Team, Nice Institute of Chemistry (ICN), University Nice Sophia Antipolis, Parc Valrose, Nice Cedex 02 F-06108, France; arnaud.cousseau@unice.fr (A.C.)

[4] Sorbonne Université, CNRS, UMR 8227, Integrative Biology of Marine Models, Station Biologique de Roscoff, CS 90074, 29688 Roscoff Cedex, France; omid.feizbakhsh@sb-roscoff.fr (O.F.)

[5] Department of Biological Sciences, Columbia University, New York, NY, 10027, USA; jcb4@columbia.edu (J.C.B.)

* Correspondence: mohamed.mehiri@univ-cotedazur.fr (M.M.); bach@sb-roscoff.fr (S.B.); Tel.: +33-492-076-154 (M.M.); Tel.: +33-298-292-391 (S.B.); Fax: +33-492-076-151 (M.M.); Fax: +33-298-292-526 (S.B.)

1. Videos

Video S1: Time-lapse imaging in living human osteosarcoma cells treated with control (DMSO) (cf. AVI video file).

Video S2: Time-lapse imaging in living human osteosarcoma cells treated with neurymenolide A (cf. AVI video file).

2. Figure

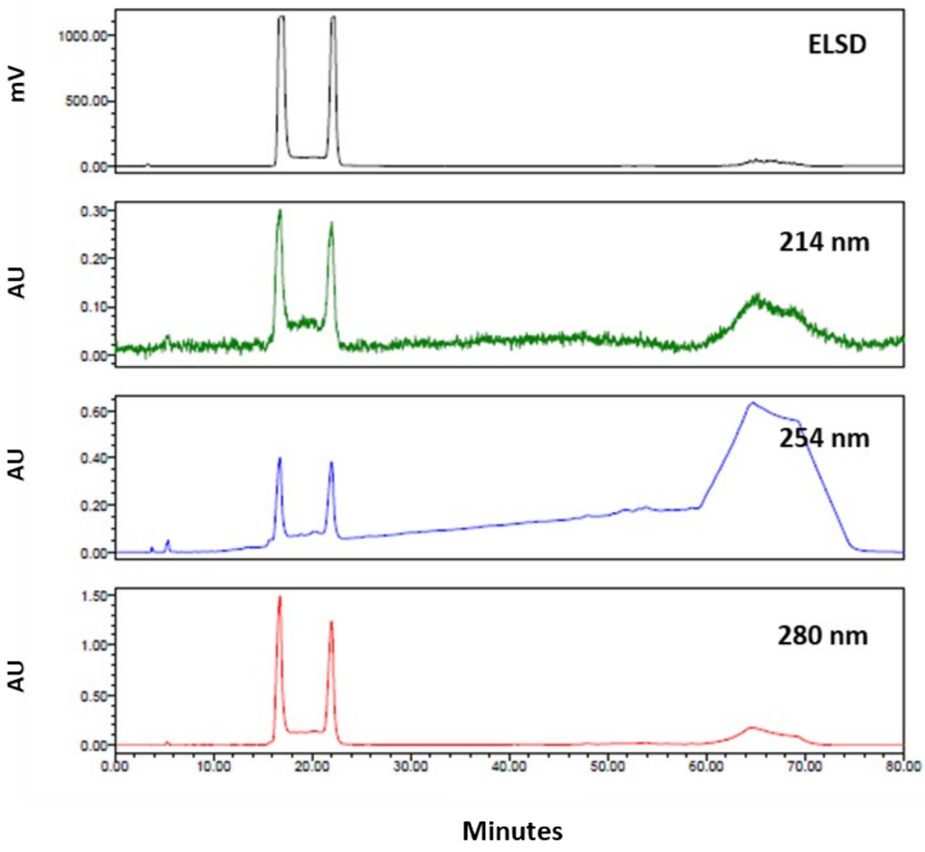

Figure S1. HPLC-UV-ELSD chromatogram of two equimolar mixtures of neurymenolide A. Interchrom Lichrospher Diol column, 250 × 4.6 mm id, 5 *μm*, gradient $C_4H_8O_2/C_6H_{14}$ 20:80 from 0 to 5 minutes and then from 20:80 to 100:0 in 55 minutes.

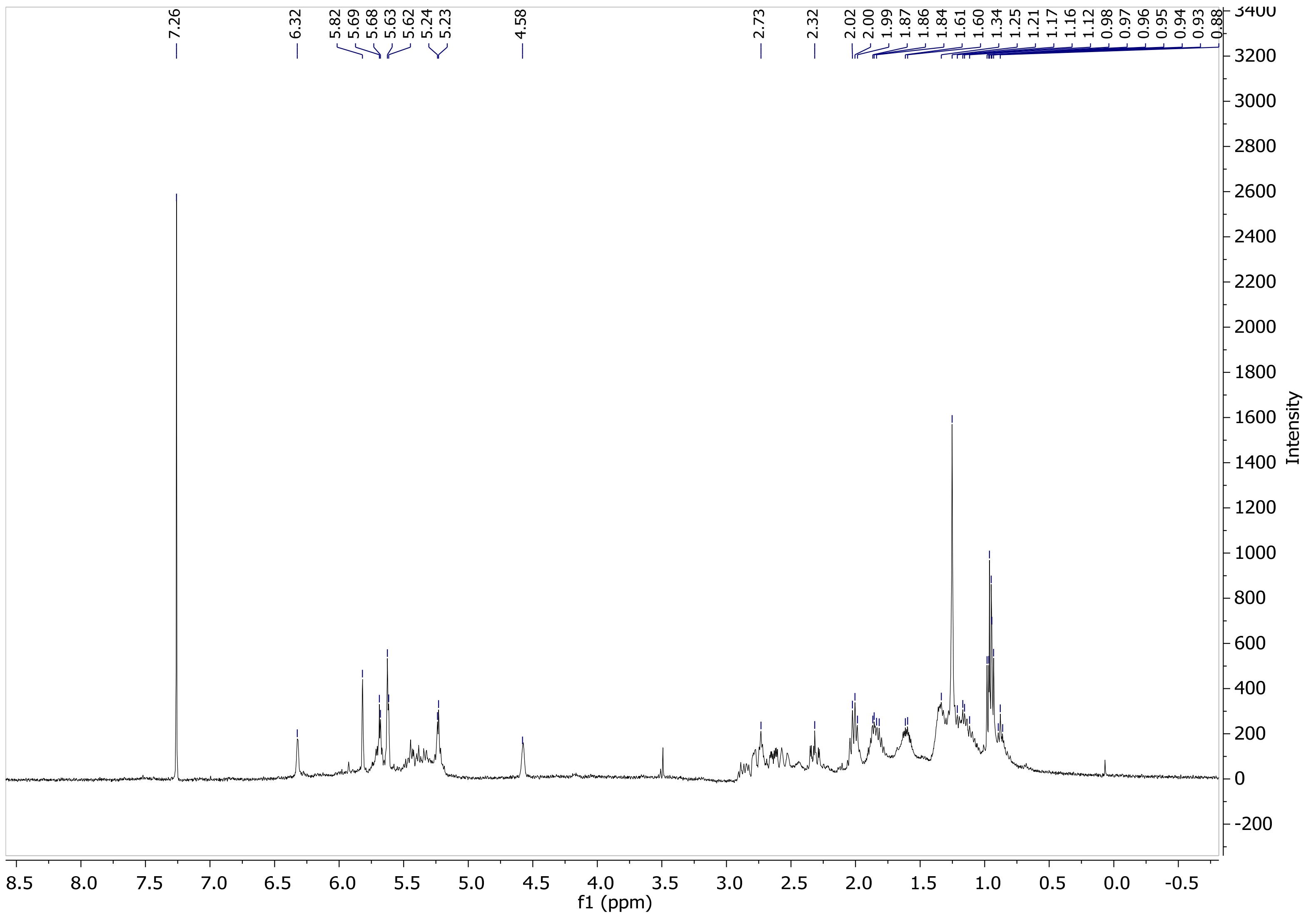

Figure S2. ^{1}H NMR spectrum of neurymenolide A in $CDCl_3$ at 400 MHz.

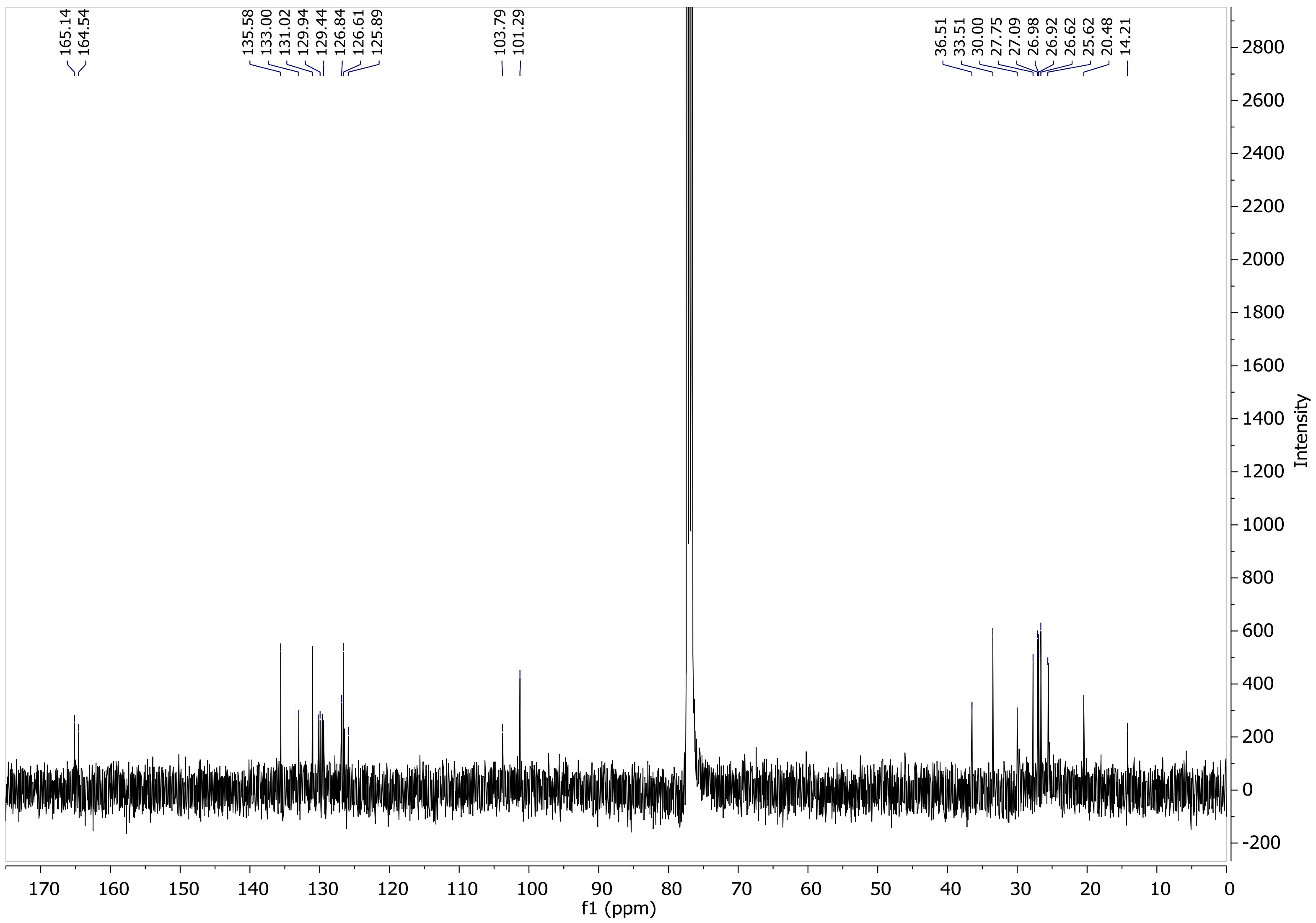

Figure S3. ^{13}C NMR spectrum of neurymenolide A in $CDCl_3$ at 100 MHz.

3. Tables

Table S1. Etotal, Boltzmann factor, ΔE and % for atropisomers of neurymenolide A.

Structure	E_{total}	ΔE	Boltzmann factor	ΔE (Kcal/mol)	%
(*P*, *S*)-C1	-1159.5253	0.00106297	0.32440	0.667023888	14.02962482
(*P*, *S*)-C2	-1159.5262	0.00021484	0.79650	0.134814164	34.44696724
(*P*, *S*)-C3	-1159.5240	0.00236237	0.08192	1.482409873	3.542869499
(*P*, *S*)-C4	-1159.5241	0.00233208	0.08459	1.463402607	3.658341442
(*P*, *S*)-C5	-1159.5264	0	1	0	43.24791869
(*P*, *S*)-C6	-1159.5229	0.00348901	0.02484	2.189387298	1.074278300
(*M*, *S*)-C1	-1159.5265	0	1	0	48.50130954
(*M*, *S*)-C2	-1159.5246	0.00194551	0.12740	1.220826218	6.179066835
(*M*, *S*)-C3	-1159.5248	0.00174408	0.15770	1.094426957	7.648656514
(*M*, *S*)-C4	-1159.5252	0.00133272	0.24380	0.836294605	11.82461926
(*M*, *S*)-C5	-1159.5251	0.00137908	0.23210	0.865385950	11.25715394
(*M*, *S*)-C6	-1159.5254	0.00113423	0.30080	0.711740223	14.58919391
(*M*, *R*)-C1	-1159.5253	0.00114690	0.29680	0.719690770	13.35348954
(*M*, *R*)-C2	-1159.5262	0.00031367	0.71730	0.196830939	32.27243278
(*M*, *R*)-C3	-1159.5240	0.00245775	0.07405	1.542261739	3.331623655
(*M*, *R*)-C4	-1159.5240	0.00245448	0.07430	1.540209783	3.342871540
(*M*, *R*)-C5	-1159.5265	0	1	0	44.99154159
(*M*, *R*)-C6	-1159.5238	0.00265340	0.06019	1.665033994	2.708040888
(*P*, *R*)-C1	-1159.5265	0	1	0	49.47311136
(*P*, *R*)-C2	-1159.5245	0.00201770	0.11800	1.266126136	5.837827141
(*P*, *R*)-C3	-1159.5247	0.00184228	0.14210	1.156048401	7.030129125
(*P*, *R*)-C4	-1159.5252	0.00133449	0.24330	0.837405297	12.03680799
(*P*, *R*)-C5	-1159.5251	0.00140245	0.22640	0.880050850	11.20071241
(*P*, *R*)-C6	-1159.5254	0.00116402	0.29150	0.730433734	14.42141196

Table S2. Coordinates (Ångstroms) for conformer (*P*, *S*)-C1 of neurymenolide A (Energy: -1159.525325 Hartree, Solvent: CH_3OH, Boltzmann %: 14.02962482).

Atom	X	Y	Z
O	-1.72766	-1.54851	-0.98034
C	-2.39691	-2.23864	-0.02307
C	-1.94315	- 2.23774	1.25275
C	-0.77682	-1.47704	1.57471
C	-0.10764	-0.71481	0.6285
C	-0.59812	-0.75247	-0.72239
O	-0.15785	-0.16149	-1.70493
C	1.1232	0.10313	1.03646
C	0.7953	1.3656	1.85208
C	0.0464	2.43429	1.54
O	-0.42743	-1.56274	2.87499
C	-0.72289	2.74276	0.27864
C	-2.09436	3.31649	0.5714
C	-3.23643	3.09939	-0.0999
C	-3.44222	2.22568	-1.31014
C	-4.63354	1.25159	-1.19242
C	-4.54694	0.29762	0.01389
C	-5.34016	-1.01196	-0.17093
C	-4.70961	-2.0303	-1.14325
C	-3.61599	-2.94263	-0.5328
C	2.11797	0.43159	-0.06305
C	3.36348	-0.05922	-0.09107
C	4.41212	0.29927	-1.11989
C	5.5955	1.00759	-0.49705
C	6.87751	0.60855	-0.46819
C	7.4795	-0.64774	-1.04207
C	8.22354	-1.47976	0.01826
H	-2.4596	-2.78964	2.03278
H	1.68667	-0.54566	1.7262
H	1.3049	1.39857	2.8175
H	0.00459	3.22685	2.29235
H	0.43366	-1.15101	3.09267
H	-0.77468	1.87986	-0.38659
H	-0.14682	3.50955	-0.27014
H	-2.12834	4.00788	1.41746
H	-4.13025	3.62349	0.25091
H	-2.53496	1.6521	-1.5295
H	-3.61433	2.87101	-2.18564
H	-4.68119	0.67423	-2.12495
H	-5.57354	1.81888	-1.13794
H	-4.91463	0.81476	0.90913
H	-3.49626	0.06965	0.22256
H	-6.34678	-0.76001	-0.53267
H	-5.48385	-1.49571	0.80547

Table S2. *Cont.*

Atom	X	Y	Z
H	-4.30232	-1.51708	-2.02257
H	-5.49117	-2.70179	-1.51886
H	-4.03798	-3.51871	0.2982
H	-3.29815	-3.66317	-1.29938
H	1.79897	1.1391	-0.82471
H	3.67869	-0.76319	0.68469
H	3.95838	0.95367	-1.87755
H	4.73958	-0.60911	-1.64076
H	5.34888	1.95462	-0.01068
H	7.59208	1.26469	0.03753
H	8.19534	-0.36787	-1.82972
H	6.71542	-1.26689	-1.52435
H	7.53682	-1.82884	0.79767
H	9.00782	-0.88803	0.50499
H	8.69806	-2.35747	-0.43454

Table S3. Coordinates (Ångstroms) for conformer (*P*, *S*)-C2 of neurymenolide A (Energy: -1159.526173 Hartree, Solvent: CH_3OH, Boltzmann %: 34.44696724).

Atom	X	Y	Z
O	-1.83664	-1.42437	-1.13109
C	-2.72289	-2.0741	-0.34057
C	-2.46053	-2.24247	0.97921
C	-1.26084	-1.68573	1.51978
C	-0.3626	-0.96966	0.74169
C	-0.64807	-0.83099	-0.65552
O	0.00767	-0.25001	-1.51725
C	0.90083	-0.40879	1.40425
C	0.63232	0.78932	2.32933
C	0.12805	2.00013	2.0478
O	-0.98093	-1.83725	2.82844
C	-0.34505	2.56085	0.72703
C	-1.65228	3.31438	0.86275
C	-2.69403	3.32025	0.0158
C	-2.82849	2.57555	-1.28743
C	-4.15558	1.8029	-1.44239
C	-4.41174	0.76755	-0.33141
C	-5.37786	-0.36241	-0.74151
C	-4.79769	-1.40797	-1.71632
C	-3.95075	-2.53097	-1.06548
C	2.07257	-0.11343	0.48698
C	3.23062	-0.78335	0.53966
C	4.44169	-0.48393	-0.30658
C	5.69694	-0.22476	0.50649
C	6.92045	-0.03902	-0.0124
C	7.25571	-0.06127	-1.48764
C	8.73361	0.22757	-1.7789
H	-3.16609	-2.76601	1.62102
H	1.23998	-1.21028	2.07183
H	0.94378	0.61837	3.36098
H	0.06757	2.70648	2.88062
H	-1.66854	-2.37402	3.29351
H	-0.39781	1.78979	-0.043
H	0.42201	3.27814	0.3841
H	-1.7228	3.94334	1.75418
H	-3.54797	3.95141	0.27891
H	-1.99347	1.87922	-1.42047
H	-2.75759	3.2992	-2.11446
H	-4.13653	1.30629	-2.42104
H	-4.99569	2.51102	-1.47726
H	-4.81708	1.27911	0.5506
H	-3.45662	0.3426	-0.00645
H	-6.26477	0.09148	-1.20527
H	-5.74059	-0.88003	0.15762

Table S3. *Cont.*

Atom	X	Y	Z
H	-4.20504	-0.91608	-2.49675
H	-5.62277	-1.9118	-2.23419
H	-4.56663	-3.09528	-0.35648
H	-3.64081	-3.23073	-1.85422
H	1.96624	0.71839	-0.2057
H	3.33227	-1.6171	1.24153
H	4.23074	0.37539	-0.96026
H	4.62779	-1.33211	-0.98566
H	5.57756	-0.18315	1.59094
H	7.74917	0.14545	0.67547
H	6.62861	0.66929	-2.01989
H	6.98421	-1.03967	-1.91237
H	9.38271	-0.51269	-1.29654
H	9.02289	1.21691	-1.40556
H	8.93804	0.20217	-2.85438

Table S4. Coordinates (Ångstroms) for conformer (*P*, *S*)-C3 of neurymenolide A (Energy: -1159.524025 Hartree, Solvent: CH_3OH, Boltzmann %: 3.542869499).

Atom	X	Y	Z
O	-1.15511	-1.73618	0.10966
C	-2.03415	-1.93551	1.12344
C	-1.95685	-1.17913	2.24364
C	-0.95992	-0.15783	2.32487
C	-0.08256	0.10317	1.28249
C	-0.18102	-0.72237	0.10899
O	0.48356	-0.65688	-0.92151
C	0.95817	1.21921	1.4233
C	0.38679	2.64281	1.29926
C	-0.29524	3.22891	0.30325
O	-0.98784	0.51945	3.49102
C	-0.72434	2.6755	-1.03334
C	-2.1498	3.05264	-1.38319
C	-3.05328	2.31452	-2.04757
C	-2.87657	0.9245	-2.60218
C	-4.00122	-0.05945	-2.21556
C	-4.20455	-0.21215	-0.69661
C	-4.86413	-1.54448	-0.2868
C	-3.95036	-2.78434	-0.37558
C	-3.02901	-3.01929	0.84795
C	2.21895	1.08967	0.58521
C	3.43647	0.9374	1.12071
C	4.73069	0.90916	0.3417
C	5.58114	-0.29933	0.66608
C	6.25583	-1.0854	-0.18801
C	6.33818	-0.97848	-1.68927
C	6.04294	-2.31314	-2.3955
H	-2.64744	-1.32608	3.06892
H	1.31674	1.14819	2.4627
H	0.63243	3.2771	2.15382
H	-0.5558	4.28029	0.45491
H	-0.24165	1.14011	3.62003
H	-0.56027	1.5994	-1.09645
H	-0.06003	3.12377	-1.79399
H	-2.44536	4.05904	-1.07537
H	-4.03245	2.76652	-2.23055
H	-1.91398	0.5059	-2.28893
H	-2.84006	0.98581	-3.701
H	-3.75696	-1.03246	-2.66123
H	-4.94715	0.25752	-2.67715
H	-4.81981	0.61912	-0.32999
H	-3.23971	-0.10315	-0.19038
H	-5.74155	-1.70964	-0.92747
H	-5.25147	-1.46261	0.73845

Table S4. *Cont.*

Atom	X	Y	Z
H	-3.33819	-2.74322	-1.28457
H	-4.57049	-3.68419	-0.4673
H	-3.63768	-3.14976	1.74955
H	-2.47718	-3.95656	0.6902
H	2.10799	1.18653	-0.49209
H	3.53258	0.83887	2.2067
H	5.3081	1.81199	0.60654
H	4.52354	0.98011	-0.73104
H	5.65805	-0.52966	1.73193
H	6.83478	-1.90753	0.24274
H	7.35465	-0.65712	-1.96406
H	5.66141	-0.20377	-2.06568
H	5.01968	-2.64846	-2.1924
H	6.72596	-3.09952	-2.0531
H	6.15927	-2.21728	-3.48063

Table S5. Coordinates (Ångstroms) for conformer (*P*, *S*)-C4 of neurymenolide A (Energy: -1159.524056 Hartree, Solvent: CH_3OH, Boltzmann %: 3.658341442).

Atom	X	Y	Z
O	-1.15106	-1.72755	-0.12639
C	-2.00628	-2.07076	0.86922
C	-1.9143	-1.46667	2.07728
C	-0.92667	-0.45214	2.27431
C	-0.0745	-0.04336	1.25841
C	-0.18853	-0.70952	-0.01178
O	0.45252	-0.49951	-1.03788
C	0.95874	1.05747	1.52055
C	0.38764	2.48624	1.54917
C	-0.29936	3.18149	0.62969
O	-0.9413	0.06663	3.51968
C	-0.74522	2.78866	-0.75719
C	-2.17696	3.19782	-1.03933
C	-3.09249	2.53357	-1.76247
C	-2.92614	1.21132	-2.4657
C	-4.03935	0.18622	-2.16203
C	-4.20419	-0.13425	-0.66489
C	-4.85925	-1.50223	-0.38688
C	-3.96958	-2.73127	-0.66648
C	-2.99425	-3.12309	0.47294
C	2.22097	1.01759	0.67548
C	3.44101	0.83091	1.19404
C	4.73167	0.87351	0.4066
C	5.59724	-0.34297	0.65006
C	5.96789	-1.28191	-0.23535
C	5.64316	-1.36076	-1.70461
C	6.90342	-1.32141	-2.58901
H	-2.58459	-1.73182	2.8898
H	1.31586	0.87932	2.54707
H	0.64478	3.02505	2.46383
H	-0.5484	4.2118	0.89965
H	-0.17946	0.64704	3.7285
H	-0.57474	1.73013	-0.95525
H	-0.09594	3.3344	-1.46549
H	-2.46597	4.16597	-0.62196
H	-4.07408	3.00203	-1.87986
H	-1.95571	0.76662	-2.22043
H	-2.91549	1.38974	-3.55228
H	-3.80489	-0.72992	-2.71936
H	-4.99713	0.54865	-2.56187
H	-4.80851	0.65123	-0.19366
H	-3.22751	-0.08178	-0.17288
H	-5.76806	-1.58193	-0.9996
H	-5.1971	-1.54046	0.65832

Table S5. *Cont.*

Atom	X	Y	Z
H	-3.40248	-2.5919	-1.59466
H	-4.60957	-3.60669	-0.83093
H	-3.56331	-3.39955	1.3675
H	-2.4332	-4.01347	0.15635
H	2.10557	1.20149	-0.38973
H	3.54298	0.63986	2.26693
H	5.29997	1.76239	0.72732
H	4.51348	1.00812	-0.65753
H	5.94464	-0.45569	1.68038
H	6.5998	-2.09429	0.13516
H	4.96055	-0.55781	-2.00176
H	5.11285	-2.30533	-1.89673
H	7.5992	-2.12492	-2.31994
H	7.43341	-0.36904	-2.47552
H	6.64382	-1.4425	-3.64666

Table S6. Coordinates (Ångstroms) for conformer (*P*, *S*)-C5 of neurymenolide A (Energy: -1159.526388 Hartree, Solvent: CH_3OH, Boltzmann %: 43.24791869).

Atom	X	Y	Z
O	-1.71651	-1.73693	-1.04677
C	-2.20037	-2.30953	0.08603
C	-1.59331	-2.08422	1.2751
C	-0.51089	-1.14875	1.34143
C	-0.05928	-0.48562	0.21414
C	-0.62684	-0.85449	-1.05647
O	-0.27513	-0.46912	-2.16634
C	1.01667	0.59971	0.24336
C	0.57661	1.86424	-0.48588
C	-0.33739	2.75056	-0.06384
O	-0.0407	-0.96759	2.59276
C	-1.13646	2.7083	1.2222
C	-2.53678	3.3027	1.14546
C	-3.51166	3.01225	0.26784
C	-3.465	2.04195	-0.88393
C	-4.56043	0.95296	-0.84877
C	-4.49642	0.0479	0.39329
C	-5.2589	-1.286	0.25311
C	-4.64559	-2.33187	-0.7006
C	-3.43233	-3.1262	-0.14914
C	2.36272	0.04702	-0.21245
C	3.31356	0.69023	-0.89846
C	4.6502	0.08366	-1.26656
C	5.81004	0.90575	-0.74946
C	6.79717	0.51362	0.0721
C	7.01382	-0.84187	0.69561
C	8.42975	-1.38894	0.44208
H	-1.96307	-2.54441	2.18687
H	1.15619	0.87907	1.30206
H	1.04	2.04097	-1.45395
H	-0.54004	3.60803	-0.70873
H	0.70135	-0.32555	2.66359
H	-0.58398	3.26396	1.99382
H	-1.19847	1.67749	1.59208
H	-2.76718	4.03961	1.91704
H	-4.46221	3.54062	0.38819
H	-2.48369	1.56082	-0.9363
H	-3.58172	2.60758	-1.82116
H	-4.45177	0.34954	-1.75912
H	-5.55358	1.42097	-0.90851
H	-4.90291	0.59294	1.25485
H	-3.44869	-0.1489	0.64296
H	-6.27917	-1.06708	-0.09256
H	-5.37008	-1.74195	1.24713

Table S6. *Cont.*

Atom	X	Y	Z
H	-4.36577	-1.86743	-1.65357
H	-5.41409	-3.07666	-0.94063
H	-3.70226	-3.61245	0.79489
H	-3.18672	-3.92056	-0.86746
H	2.558	-0.97832	0.11358
H	3.15749	1.71938	-1.22759
H	4.72306	0.04292	-2.36594
H	4.69804	-0.94924	-0.9081
H	5.82266	1.94283	-1.09479
H	7.55196	1.26063	0.33486
H	6.27292	-1.56388	0.33661
H	6.8621	-0.75811	1.78266
H	9.19301	-0.69562	0.81463
H	8.6088	-1.53793	-0.62876
H	8.57532	-2.35029	0.94696

Table S7. Coordinates (Ångstroms) for conformer (*P*, *S*)-C6 of neurymenolide A (Energy: -1159.522899 Hartree, Solvent: CH_3OH, Boltzmann %: 1.0742783).

Atom	X	Y	Z
O	-1.16455	-1.75778	0.05492
C	-2.0693	-1.98633	1.03864
C	-2.0253	-1.25902	2.17993
C	-1.02997	-0.24267	2.31817
C	-0.12964	0.05183	1.30411
C	-0.20083	-0.73632	0.10351
O	0.48026	-0.6315	-0.91329
C	0.90125	1.16954	1.49606
C	0.32519	2.59315	1.3965
C	-0.35821	3.19663	0.41187
O	-1.07926	0.39096	3.50835
C	-0.78833	2.66975	-0.93496
C	-2.20705	3.07045	-1.28697
C	-3.09984	2.36951	-2.00373
C	-2.91352	1.00852	-2.62309
C	-4.02657	-0.00392	-2.27744
C	-4.23174	-0.21684	-0.76624
C	-4.87842	-1.57074	-0.40996
C	-3.94274	-2.79171	-0.53119
C	-3.05198	-3.06542	0.70669
C	2.17351	1.06778	0.6717
C	3.38246	0.87413	1.21295
C	4.68185	0.8545	0.44383
C	5.50674	-0.38438	0.71253
C	6.2214	-1.07998	-0.18553
C	6.35687	-0.78696	-1.66409
C	6.89053	-1.98346	-2.46359
H	-2.73709	-1.43049	2.98216
H	1.2441	1.07292	2.5384
H	0.57344	3.21368	2.26035
H	-0.61795	4.24519	0.58311
H	-0.32277	0.98776	3.68281
H	-0.63987	1.59274	-1.01335
H	-0.1135	3.11909	-1.68552
H	-2.50638	4.05968	-0.93105
H	-4.07616	2.83095	-2.17805
H	-1.94531	0.58621	-2.33302
H	-2.88212	1.12083	-3.71791
H	-3.76782	-0.95645	-2.7578
H	-4.97535	0.31712	-2.73009
H	-4.85381	0.59463	-0.36837
H	-3.26864	-0.1202	-0.25417
H	-5.74739	-1.72581	-1.06418
H	-5.27597	-1.52912	0.61371

Table S7. *Cont.*

Atom	X	Y	Z
H	-3.30381	-2.69498	-1.41733
H	-4.5416	-3.69714	-0.68549
H	-3.68202	-3.22617	1.58833
H	-2.49303	-3.99556	0.53314
H	2.07788	1.22008	-0.40078
H	3.46587	0.72685	2.29447
H	5.27736	1.73318	0.74807
H	4.48524	0.97396	-0.62731
H	5.52603	-0.71717	1.75325
H	6.78402	-1.94334	0.17957
H	7.03248	0.06971	-1.80884
H	5.38947	-0.47406	-2.07817
H	6.22119	-2.84724	-2.37598
H	7.87874	-2.29049	-2.10133
H	6.98498	-1.73812	-3.52661

Table S8. Coordinates (Ångstroms) for conformer (*M*, *S*)-C1 of neurymenolide A (Energy: -1159.526514 Hartree, Solvent: CH_3OH, Boltzmann %: 48.50130954).

Atom	X	Y	Z
C	-1.02376	-1.76379	-0.89745
C	-1.76507	-2.21853	0.14226
C	-0.69925	-0.65362	1.65752
C	0.01213	-0.0752	0.5516
C	-0.12636	-0.6702	-0.69125
O	0.52607	-0.28404	-1.80616
C	0.85062	1.16548	0.83226
C	0.53659	2.3277	-0.11668
C	-0.56977	3.08755	-0.09981
O	-0.63433	-0.31629	2.83961
C	-1.73839	2.96451	0.8573
C	-3.09091	3.37015	0.28821
C	-3.76554	2.76323	-0.70156
C	-3.32292	1.57376	-1.512
C	-4.26317	0.35029	-1.44352
C	-4.38083	-0.2634	-0.03809
C	-4.96939	-1.68822	-0.00308
C	-4.14014	-2.80841	-0.66379
C	-2.85156	-3.25261	0.07832
C	2.34912	0.90965	0.89515
C	2.96825	-0.27497	0.83607
C	4.46115	-0.46817	0.9736
C	5.05512	-1.23358	-0.18799
C	6.10851	-0.89335	-0.94802
C	6.97989	0.33213	-0.84694
C	8.46074	-0.01759	-0.60887
H	0.55947	1.48802	1.84077
H	1.30723	2.57096	-0.85323
H	-0.63991	3.89765	-0.82873
H	-1.53427	3.60697	1.72613
H	-1.79234	1.94479	1.25313
H	-3.5569	4.239	0.75631
H	-4.74165	3.17555	-0.97316
H	-2.31576	1.26587	-1.2129
H	-3.25017	1.88512	-2.56525
H	-3.87587	-0.3966	-2.14731
H	-5.26271	0.62588	-1.80931
H	-5.01075	0.38464	0.58478
H	-3.39685	-0.25562	0.4419
H	-5.95198	-1.66834	-0.49561
H	-5.16156	-1.96704	1.04255
H	-3.88168	-2.53946	-1.69526
H	-4.77336	-3.70167	-0.73485
H	-3.09715	-3.5574	1.10161

Table S8. *Cont.*

Atom	X	Y	Z
H	-2.45101	-4.13428	-0.43708
H	2.94762	1.81387	1.03899
H	2.38365	-1.18701	0.69828
H	4.64536	-1.04791	1.89385
H	4.95085	0.50078	1.11123
H	4.55356	-2.17823	-0.41407
H	6.39407	-1.58929	-1.74218
H	6.63089	1.00152	-0.05376
H	6.90351	0.89753	-1.78778
H	8.84426	-0.66993	-1.40218
H	8.59292	-0.53965	0.34542
H	9.07886	0.88687	-0.59018
H	1.13313	0.46833	-1.67229
O	-1.58423	-1.70138	1.38572
H	-1.14201	-2.18403	-1.89267

Table S9. Coordinates (Ångstroms) for conformer (*M*, *S*)-C2 of neurymenolide A (Energy: -1159.524569 Hartree, Solvent: CH_3OH, Boltzmann %: 6.179066835).

Atom	X	Y	Z
C	-0.59132	-2.05229	-0.51815
C	-1.52747	-2.31006	0.42971
C	-0.81183	-0.40006	1.74846
C	0.09182	-0.03211	0.69769
C	0.24195	-0.90355	-0.36698
O	1.14383	-0.71475	-1.35362
C	0.83971	1.29947	0.80341
C	0.44987	2.2491	-0.32489
C	-0.65695	3.00406	-0.39617
O	-0.99466	0.18321	2.81706
C	-1.76437	3.13087	0.62508
C	-3.17259	3.26655	0.05899
C	-3.77571	2.52059	-0.88174
C	-3.22182	1.34659	-1.64736
C	-4.09726	0.07538	-1.57229
C	-4.26011	-0.47066	-0.14381
C	-4.75525	-1.92772	-0.05101
C	-3.79928	-3.03271	-0.54452
C	-2.58505	-3.37136	0.36514
C	2.33733	1.05082	0.92905
C	3.33504	1.66387	0.27579
C	4.79975	1.36567	0.50631
C	5.50896	0.94539	-0.76185
C	6.21911	-0.17487	-0.97069
C	6.49129	-1.30313	-0.00932
C	6.0201	-2.66479	-0.55176
H	0.51838	1.73397	1.75829
H	1.12851	2.29842	-1.17707
H	-0.78177	3.62143	-1.28852
H	-1.56868	4.03536	1.21997
H	-1.72802	2.30078	1.34046
H	-3.76233	4.07246	0.49984
H	-4.80607	2.78724	-1.13639
H	-2.21248	1.1063	-1.29946
H	-3.1264	1.63105	-2.70669
H	-3.63412	-0.68203	-2.21724
H	-5.08814	0.27942	-2.00297
H	-4.96802	0.16665	0.4015
H	-3.31104	-0.36564	0.38894
H	-5.68766	-2.01359	-0.62718
H	-5.02539	-2.14387	0.99216
H	-3.43292	-2.80576	-1.5535
H	-4.37588	-3.96184	-0.63501
H	-2.93068	-3.58381	1.38325

Table S9. *Cont.*

Atom	X	Y	Z
H	-2.12022	-4.28542	-0.0225
H	2.59372	0.2853	1.66682
H	3.12255	2.44169	-0.46028
H	4.90542	0.61363	1.29415
H	5.27847	2.28582	0.88019
H	5.42032	1.65217	-1.59087
H	6.66214	-0.30555	-1.96228
H	6.0253	-1.11515	0.96363
H	7.57521	-1.35704	0.17259
H	6.49159	-2.89024	-1.51554
H	4.93478	-2.67383	-0.70114
H	6.27458	-3.47254	0.14329
H	1.77706	-0.01255	-1.11792
O	-1.59206	-1.54528	1.55119
H	-0.5006	-2.67858	-1.40194

Table S10. Coordinates (Ångstroms) for conformer (*M*, *S*)-C3 of neurymenolide A (Energy: -1159.52477 Hartree, Solvent: CH_3OH, Boltzmann %: 7.648656514).

Atom	X	Y	Z
C	-0.6612	-2.09966	-0.55545
C	-1.61613	-2.34972	0.37555
C	-0.8923	-0.46114	1.72074
C	0.03219	-0.09939	0.6868
C	0.18697	-0.96527	-0.38161
O	1.10713	-0.77888	-1.35218
C	0.79131	1.22432	0.80635
C	0.39082	2.19229	-0.30238
C	-0.706	2.96373	-0.34289
O	-1.0814	0.11602	2.79136
C	-1.78692	3.10043	0.70453
C	-3.20689	3.25793	0.17548
C	-3.83769	2.54745	-0.77466
C	-3.31468	1.39474	-1.59319
C	-4.21102	0.1366	-1.55099
C	-4.35787	-0.4626	-0.14201
C	-4.85304	-1.92206	-0.1004
C	-3.89384	-3.0088	-0.62777
C	-2.69191	-3.38979	0.28211
C	2.28921	0.96343	0.90209
C	3.27881	1.58266	0.24174
C	4.74561	1.26917	0.4361
C	5.42592	0.8836	-0.85877
C	6.05432	-0.26814	-1.14405
C	6.25711	-1.46902	-0.25666
C	7.74619	-1.7883	-0.02718
H	0.48808	1.64552	1.773
H	1.04953	2.23576	-1.17034
H	-0.84213	3.58863	-1.22831
H	-1.56468	3.99986	1.29803
H	-1.74487	2.26737	1.41629
H	-3.78189	4.05119	0.65709
H	-4.87187	2.83007	-0.99421
H	-2.30414	1.12515	-1.27136
H	-3.2313	1.71695	-2.64272
H	-3.77475	-0.60383	-2.23371
H	-5.20594	0.37548	-1.95358
H	-5.05835	0.1539	0.43599
H	-3.40255	-0.37839	0.38293
H	-5.78208	-1.98838	-0.68451
H	-5.12891	-2.17415	0.93314
H	-3.51351	-2.73956	-1.62123
H	-4.47172	-3.93128	-0.76559
H	-3.04826	-3.61896	1.29289

Table S10. *Cont.*

Atom	X	Y	Z
H	-2.24083	-4.30277	-0.1237
H	2.55397	0.18282	1.62093
H	3.05733	2.37602	-0.47481
H	4.86036	0.49304	1.1987
H	5.23808	2.17462	0.82668
H	5.38471	1.64579	-1.64106
H	6.49107	-0.36188	-2.14257
H	5.78406	-2.34047	-0.7333
H	5.75847	-1.3367	0.70922
H	8.24595	-0.97145	0.50533
H	8.26918	-1.9357	-0.97933
H	7.86354	-2.70246	0.56523
H	1.74997	-0.09288	-1.09136
O	-1.68479	-1.59446	1.50318
H	-0.56789	-2.71953	-1.44349

Table S11. Coordinates (Ångstroms) for conformer (*M*, *S*)-C4 of neurymenolide A (Energy: -1159.525181 Hartree, Solvent: CH_3OH, Boltzmann %: 11.82461926).

Atom	X	Y	Z
C	-1.36206	-2.1439	-0.6327
C	-2.39478	-2.19553	0.24583
C	-1.27671	-0.64913	1.74994
C	-0.23275	-0.48521	0.78117
C	-0.25889	-1.28559	-0.34701
O	0.72348	-1.28543	-1.27355
C	0.8544	0.56065	1.03992
C	0.82044	1.67722	-0.00069
C	-0.04032	2.7049	-0.05578
O	-1.36813	-0.10709	2.85081
C	-1.15906	3.03723	0.90477
C	-2.43399	3.57521	0.26614
C	-3.12787	3.07396	-0.76951
C	-2.82731	1.84465	-1.58751
C	-4.01561	0.86627	-1.71892
C	-4.50687	0.31157	-0.3708
C	-5.37126	-0.96105	-0.4644
C	-4.67809	-2.25183	-0.9448
C	-3.6893	-2.92676	0.04544
C	2.21054	-0.11935	1.18555
C	3.3803	0.24364	0.63814
C	4.68676	-0.48473	0.86575
C	5.23526	-1.08153	-0.41233
C	6.32773	-0.70367	-1.0959
C	7.30068	0.39908	-0.76936
C	8.74373	-0.11516	-0.61423
H	0.61728	0.98736	2.02306
H	1.55684	1.61674	-0.80247
H	0.07635	3.40603	-0.88511
H	-0.79088	3.81395	1.59142
H	-1.39182	2.17551	1.54124
H	-2.8294	4.47965	0.73232
H	-4.02829	3.61806	-1.07041
H	-1.96874	1.31134	-1.16851
H	-2.5328	2.15898	-2.60079
H	-3.69448	0.04477	-2.37173
H	-4.84947	1.36083	-2.23765
H	-5.09218	1.08642	0.14082
H	-3.64541	0.12871	0.27786
H	-6.21074	-0.76211	-1.14596
H	-5.82179	-1.15563	0.51906
H	-4.16313	-2.0825	-1.89865
H	-5.45767	-2.99611	-1.15051
H	-4.17058	-3.06041	1.02085

Table S11. *Cont.*

Atom	X	Y	Z
H	-3.45311	-3.92434	-0.34347
H	2.19577	-0.99458	1.8413
H	3.44371	1.11692	-0.01315
H	4.52906	-1.28566	1.60081
H	5.41019	0.21409	1.30291
H	4.64748	-1.90989	-0.81523
H	6.56186	-1.26159	-2.0072
H	7.00191	0.93598	0.13746
H	7.27986	1.13602	-1.58607
H	9.07337	-0.64262	-1.51704
H	8.82507	-0.81131	0.22798
H	9.43762	0.71412	-0.43793
H	1.50473	-0.80014	-0.95029
O	-2.32844	-1.51406	1.41926
H	-1.38569	-2.7115	-1.55936

Table S12. Coordinates (Ångstroms) for conformer (*M*, *S*)-C5 of neurymenolide A (Energy: -1159.525135 Hartree, Solvent: CH_3OH, Boltzmann %: 11.25715394).

Atom	X	Y	Z
C	-1.43525	-2.13137	-0.77761
C	-2.47831	-2.18667	0.08835
C	-1.29947	-0.79957	1.69712
C	-0.24198	-0.62011	0.74553
C	-0.29524	-1.34636	-0.43089
O	0.69383	-1.33493	-1.35002
C	0.88424	0.36367	1.07232
C	0.9064	1.53629	0.09576
C	0.09525	2.60479	0.10019
O	-1.37305	-0.32768	2.83146
C	-1.0068	2.93097	1.08191
C	-2.24338	3.59386	0.48809
C	-2.9622	3.22082	-0.58391
C	-2.7377	2.04702	-1.50202
C	-3.96912	1.12684	-1.66017
C	-4.43439	0.4943	-0.33767
C	-5.36846	-0.72284	-0.48693
C	-4.76383	-2.00938	-1.08413
C	-3.80986	-2.82694	-0.16918
C	2.21065	-0.37629	1.19213
C	3.40474	-0.01294	0.70103
C	4.68218	-0.7945	0.91466
C	5.29149	-1.27262	-0.38555
C	6.49576	-0.96598	-0.89507
C	7.55612	-0.06355	-0.31926
C	7.95775	1.06481	-1.28712
H	0.65175	0.74361	2.07557
H	1.64285	1.48658	-0.70707
H	0.24664	3.34771	-0.68605
H	-0.59551	3.62667	1.82835
H	-1.29642	2.03803	1.64806
H	-2.58717	4.47868	1.02707
H	-3.82945	3.83907	-0.83533
H	-1.8868	1.45005	-1.15926
H	-2.46803	2.42739	-2.49937
H	-3.70515	0.34407	-2.38261
H	-4.7997	1.69052	-2.10901
H	-4.95615	1.2558	0.25605
H	-3.55736	0.22001	0.25552
H	-6.21943	-0.43133	-1.11924
H	-5.79462	-0.96472	0.49689
H	-4.24972	-1.79302	-2.02895
H	-5.59051	-2.68466	-1.33838
H	-4.29569	-3.02419	0.79313

Table S12. *Cont.*

Atom	X	Y	Z
H	-3.62922	-3.79505	-0.65096
H	2.15051	-1.29549	1.78183
H	3.51053	0.90475	0.11957
H	4.46758	-1.66132	1.55547
H	5.3949	-0.16489	1.46114
H	4.65756	-1.95117	-0.96148
H	6.75899	-1.42065	-1.85454
H	8.44958	-0.66716	-0.09969
H	7.23349	0.36853	0.6339
H	7.10926	1.72668	-1.49371
H	8.30517	0.6583	-2.24417
H	8.76728	1.67127	-0.86639
H	1.49221	-0.90131	-0.99765
O	-2.38717	-1.5891	1.30556
H	-1.47779	-2.63463	-1.74015

Table S13. Coordinates (Ångstroms) for conformer (*M*, *S*)-C6 of neurymenolide A (Energy: -1159.52538 Hartree, Solvent: CH_3OH, Boltzmann %: 14.58919391).

Atom	X	Y	Z
C	-1.67766	-2.13665	-0.85547
C	-2.60845	-2.17613	0.13077
C	-1.21304	-0.82522	1.58813
C	-0.27186	-0.66536	0.51778
C	-0.4844	-1.38082	-0.64728
O	0.38214	-1.38678	-1.6824
C	0.91397	0.28061	0.7194
C	0.86122	1.46616	-0.2414
C	0.08601	2.55652	-0.13991
O	-1.13806	-0.3579	2.72424
C	-0.89871	2.90015	0.95473
C	-2.17996	3.58291	0.49131
C	-3.01893	3.20763	-0.48859
C	-2.91785	2.01105	-1.39935
C	-4.18844	1.13286	-1.43321
C	-4.55239	0.52784	-0.06712
C	-5.5262	-0.66567	-0.11969
C	-5.01398	-1.96804	-0.76793
C	-3.97295	-2.79279	0.03805
C	2.22507	-0.49465	0.67692
C	3.37539	-0.1258	0.09508
C	4.65192	-0.9359	0.14691
C	5.73365	-0.22381	0.93079
C	6.89235	0.27459	0.46939
C	7.42907	0.24699	-0.93825
C	8.78335	-0.4788	-1.03934
H	0.81241	0.65741	1.74563
H	1.51129	1.40891	-1.11529
H	0.17495	3.30532	-0.93011
H	-0.39932	3.58913	1.65193
H	-1.14106	2.01189	1.54974
H	-2.44219	4.48556	1.0463
H	-3.89522	3.84046	-0.65895
H	-2.06166	1.38958	-1.11931
H	-2.72256	2.36505	-2.42348
H	-4.01577	0.33436	-2.16582
H	-5.03721	1.71939	-1.81335
H	-5.00096	1.30904	0.55992
H	-3.63456	0.23667	0.45142
H	-6.42686	-0.35531	-0.66855
H	-5.86035	-0.89499	0.90196
H	-4.61096	-1.76923	-1.76874
H	-5.87571	-2.63093	-0.91615
H	-4.34503	-2.97254	1.05304

Table S13. *Cont.*

Atom	X	Y	Z
H	-3.86753	-3.76909	-0.44989
H	2.19851	-1.44427	1.21878
H	3.44976	0.82683	-0.43227
H	4.99159	-1.14139	-0.87514
H	4.44259	-1.90601	0.61814
H	5.51514	-0.10055	1.99424
H	7.54877	0.76132	1.19658
H	7.56392	1.28392	-1.28067
H	6.71262	-0.21375	-1.62675
H	8.68555	-1.53451	-0.7628
H	9.52449	-0.02563	-0.37057
H	9.17842	-0.43003	-2.06008
H	1.22956	-0.97221	-1.43809
O	-2.35872	-1.585	1.32881
H	-1.84854	-2.63303	-1.80721

Table S14. Coordinates (Ångstroms) for conformer (*M*, *R*)-C1 of neurymenolide A (Energy: -1159.525335 Hartree, Solvent: CH_3OH, Boltzmann %: 13.35348954).

Atom	X	Y	Z
O	1.72924	-1.55272	-0.98038
C	2.39956	-2.23833	-0.02073
C	1.94829	-2.23068	1.25588
C	0.78286	-1.46758	1.57637
C	0.11023	-0.71322	0.62603
C	0.59899	-0.75666	-0.72538
O	0.15625	-0.17108	-1.71021
C	-1.12209	0.10284	1.032
C	-0.798	1.36529	1.84884
C	-0.04924	2.43425	1.53769
O	0.43836	-1.54376	2.87817
C	0.71948	2.74265	0.27584
C	2.09125	3.31712	0.56624
C	3.23273	3.09974	-0.10607
C	3.43825	2.22373	-1.31465
C	4.62856	1.24889	-1.19426
C	4.54063	0.29825	0.01452
C	5.33868	-1.00914	-0.16446
C	4.71413	-2.03132	-1.1363
C	3.61888	-2.94326	-0.5283
C	-2.11735	0.43121	-0.06677
C	-3.36158	-0.06298	-0.09455
C	-4.41301	0.2968	-1.12
C	-5.59558	1.00469	-0.49477
C	-6.87774	0.6056	-0.46381
C	-7.48193	-0.65021	-1.03745
C	-8.22477	-1.48312	0.02338
H	2.46663	-2.77832	2.03764
H	-1.68495	-0.54719	1.72111
H	-1.30968	1.39769	2.81317
H	-0.00858	3.22756	2.28938
H	-0.42222	-1.13136	3.0974
H	0.77087	1.87937	-0.38873
H	0.14239	3.50878	-0.27262
H	2.12553	4.01036	1.41077
H	4.12628	3.62589	0.24233
H	2.53039	1.65096	-1.53348
H	3.61179	2.86755	-2.19094
H	4.67609	0.66901	-2.12517
H	5.56899	1.81553	-1.14106
H	4.90287	0.81871	0.90994
H	3.48988	0.06768	0.21916
H	6.34543	-0.75472	-0.52411
H	5.48134	-1.49023	0.81331

Table S14. *Cont.*

Atom	X	Y	Z
H	4.30993	-1.52132	-2.01898
H	5.49825	-2.70279	-1.5065
H	4.03882	-3.51991	0.30319
H	3.30222	-3.66317	-1.29596
H	-1.80052	1.14143	-0.82678
H	-3.67311	-0.77108	0.67891
H	-3.96061	0.95175	-1.8781
H	-4.74183	-0.61084	-1.6406
H	-5.34836	1.95238	-0.01002
H	-7.59132	1.2627	0.042
H	-8.19937	-0.36921	-1.82311
H	-6.71968	-1.26928	-1.52211
H	-7.53684	-1.83503	0.80034
H	-9.00663	-0.89073	0.51306
H	-8.70212	-2.35901	-0.42978

Table S15. Coordinates (Ångstroms) for conformer (*M*, *R*)-C2 of neurymenolide A (Energy: -1159.52616825 Hartree, Solvent: CH_3OH, Boltzmann %: 32.27243278).

Atom	X	Y	Z
O	1.83683	-1.41973	-1.13815
C	2.72178	-2.07577	-0.35134
C	2.4578	-2.25346	0.9669
C	1.25786	-1.69979	1.51016
C	0.36144	-0.97672	0.73654
C	0.64905	-0.82765	-0.65923
O	-0.00423	-0.23845	-1.5172
C	-0.90144	-0.41804	1.40224
C	-0.63184	0.77658	2.33129
C	-0.12685	1.98807	2.05386
O	0.97682	-1.85985	2.81761
C	0.34737	2.5529	0.73522
C	1.65168	3.31067	0.87579
C	2.69446	3.32478	0.03022
C	2.83403	2.58708	-1.27638
C	4.16178	1.81516	-1.42943
C	4.41058	0.77165	-0.32453
C	5.37562	-0.358	-0.73801
C	4.7958	-1.39637	-1.7206
C	3.95126	-2.52567	-1.07777
C	-2.07367	-0.11859	0.48682
C	-3.23212	-0.78807	0.53689
C	-4.44296	-0.48434	-0.30811
C	-5.69694	-0.22236	0.50598
C	-6.9202	-0.03293	-0.01218
C	-7.25598	-0.0523	-1.48735
C	-8.73319	0.24064	-1.77785
H	3.16266	-2.78135	1.60594
H	-1.24022	-1.22195	2.06717
H	-0.94473	0.60282	3.36206
H	-0.06708	2.69182	2.88894
H	1.66334	-2.40086	3.27936
H	0.40456	1.78346	-0.03619
H	-0.42127	3.26824	0.39174
H	1.71927	3.93479	1.77085
H	3.54627	3.95664	0.2985
H	1.99975	1.89117	-1.41627
H	2.7664	3.31485	-2.1001
H	4.14809	1.32566	-2.4118
H	5.00244	2.52309	-1.45417
H	4.81267	1.27599	0.56312
H	3.45288	0.34666	-0.0073
H	6.26506	0.09639	-1.19645
H	5.73419	-0.88158	0.15936

Table S15. *Cont.*

Atom	X	Y	Z
H	4.2008	-0.89865	-2.49557
H	5.62059	-1.89457	-2.24443
H	4.56776	-3.09281	-0.37153
H	3.64388	-3.22119	-1.87128
H	-1.96731	0.71613	-0.20236
H	-3.33418	-1.62482	1.23514
H	-4.22987	0.37499	-0.96114
H	-4.63204	-1.33137	-0.98787
H	-5.57689	-0.1824	1.59042
H	-7.74821	0.15291	0.67621
H	-6.62712	0.67734	-2.01884
H	-6.9871	-1.03087	-1.91342
H	-9.38402	-0.49886	-1.2966
H	-9.02004	1.23008	-1.40285
H	-8.93784	0.21749	-2.85337

Table S16. Coordinates (Ångstroms) for conformer (*M*, *R*)-C3 of neurymenolide A (Energy: -1159.524024 Hartree, Solvent: CH_3OH, Boltzmann %: 3.331623655).

Atom	X	Y	Z
O	1.15524	-1.73569	0.10942
C	2.03405	-1.93546	1.12331
C	1.95661	-1.17943	2.24375
C	0.95979	-0.15804	2.32512
C	0.0826	0.10332	1.28267
C	0.18118	-0.72185	0.10894
O	-0.48314	-0.65582	-0.92169
C	-0.95815	1.21931	1.42356
C	-0.38682	2.64291	1.29937
C	0.29499	3.22894	0.30317
O	0.98753	0.51879	3.49153
C	0.72389	2.67539	-1.03342
C	2.14925	3.05264	-1.38355
C	3.05277	2.31449	-2.04784
C	2.8762	0.92435	-2.60217
C	4.00107	-0.05934	-2.21554
C	4.20438	-0.21204	-0.69659
C	4.86399	-1.54435	-0.2868
C	3.95028	-2.78424	-0.37574
C	3.02882	-3.01928	0.84769
C	-2.21887	1.08971	0.58541
C	-3.43643	0.93752	1.12082
C	-4.73055	0.90904	0.34168
C	-5.5809	-0.29951	0.66609
C	-6.2554	-1.08572	-0.18801
C	-6.33758	-0.9789	-1.68928
C	-6.04181	-2.31349	-2.39541
H	2.64703	-1.32674	3.06911
H	-1.31674	1.14831	2.46295
H	-0.6324	3.27729	2.1539
H	0.55551	4.28036	0.45465
H	0.24152	1.13965	3.62054
H	0.55991	1.59925	-1.09635
H	0.05941	3.1235	-1.79401
H	2.44471	4.05915	-1.07596
H	4.03187	2.7666	-2.23099
H	1.91373	0.50564	-2.28869
H	2.8395	0.98546	-3.701
H	3.75702	-1.0324	-2.66122
H	4.94694	0.25784	-2.67712
H	4.81961	0.61925	-0.32994
H	3.23953	-0.10309	-0.19038
H	5.74147	-1.70944	-0.92741
H	5.25125	-1.46253	0.73849

Table S16. *Cont.*

Atom	X	Y	Z
H	3.3382	-2.74308	-1.28478
H	4.57045	-3.68406	-0.46745
H	3.6374	-3.14999	1.74931
H	2.4769	-3.95647	0.68973
H	-2.10781	1.18638	-0.49191
H	-3.53264	0.83917	2.20682
H	-5.30809	1.81184	0.6063
H	-4.52326	0.97985	-0.73105
H	-5.65782	-0.52983	1.73195
H	-6.83424	-1.90794	0.24272
H	-7.35411	-0.65786	-1.96423
H	-5.66098	-0.20401	-2.06562
H	-5.01848	-2.64849	-2.19215
H	-6.72464	-3.10008	-2.05309
H	-6.15801	-2.21772	-3.48057

Table S17. Coordinates (Ångstroms) for conformer (*M*, *R*)-C4 of neurymenolide A (Energy: -1159.524027 Hartree, Solvent: CH_3OH, Boltzmann %: 3.34287154).

Atom	X	Y	Z
O	1.14594	-1.72578	-0.12472
C	1.99901	-2.06809	0.87308
C	1.9072	-1.45989	2.07907
C	0.92149	-0.44269	2.27204
C	0.06945	-0.03699	1.25483
C	0.18273	-0.7081	-0.0128
O	-0.45679	-0.49961	-1.04016
C	-0.9626	1.06598	1.51277
C	-0.39138	2.49494	1.53456
C	0.30011	3.18494	0.61444
O	0.9373	0.08111	3.51526
C	0.75315	2.78504	-0.76807
C	2.18651	3.19231	-1.04483
C	3.1047	2.52576	-1.76248
C	2.94031	1.20239	-2.46402
C	4.05137	0.17695	-2.15373
C	4.20927	-0.14127	-0.65534
C	4.85854	-1.51084	-0.37182
C	3.96672	-2.73781	-0.65339
C	2.9843	-3.12471	0.48169
C	-2.22575	1.02275	0.66921
C	-3.44508	0.83707	1.18983
C	-4.73696	0.87773	0.4042
C	-5.60145	-0.33899	0.65058
C	-5.96673	-1.28307	-0.23159
C	-5.63748	-1.36727	-1.69953
C	-6.89512	-1.33069	-2.58781
H	2.57608	-1.72394	2.89309
H	-1.31872	0.8927	2.54049
H	-0.6533	3.03946	2.44447
H	0.54725	4.2169	0.8798
H	0.17602	0.66302	3.7218
H	0.58293	1.72577	-0.96219
H	0.10793	3.32799	-1.48221
H	2.47439	4.16131	-0.62865
H	4.08713	2.99326	-1.87663
H	1.96847	0.75891	-2.22219
H	2.93452	1.37886	-3.55095
H	3.81861	-0.73978	-2.71074
H	5.01119	0.53798	-2.5499
H	4.81474	0.6429	-0.18339
H	3.23105	-0.08423	-0.16691
H	5.77015	-1.59444	-0.97985
H	5.19103	-1.54816	0.67514

Table S17. *Cont.*

Atom	X	Y	Z
H	3.40488	-2.59894	-1.58479
H	4.60511	-3.61537	-0.81258
H	3.54806	-3.40323	1.37895
H	2.42086	-4.01277	0.1629
H	-2.11162	1.20352	-0.39669
H	-3.54556	0.64889	2.26337
H	-5.30527	1.76667	0.7246
H	-4.52053	1.01107	-0.66044
H	-5.95246	-0.44732	1.68013
H	-6.59828	-2.09504	0.14043
H	-4.95387	-0.56543	-1.99736
H	-5.10677	-2.31259	-1.88671
H	-7.59185	-2.13314	-2.31806
H	-7.42519	-0.37784	-2.47898
H	-6.63246	-1.45537	-3.64428

Table S18. Coordinates (Ångstroms) for conformer (*M*, *R*)-C5 of neurymenolide A (Energy: -1159.526482 Hartree, Solvent: CH_3OH, Boltzmann %: 44.99154159).

Atom	X	Y	Z
O	1.71779	-1.73853	-1.05181
C	2.19653	-2.312	0.08295
C	1.58327	-2.08852	1.26911
C	0.50016	-1.1534	1.3309
C	0.05404	-0.48945	0.202
C	0.62727	-0.8582	-1.06604
O	0.27737	-0.4758	-2.17771
C	-1.02065	0.59654	0.22582
C	-0.57453	1.85807	-0.50616
C	0.33203	2.74989	-0.08008
O	0.02175	-0.97524	2.57969
C	1.11159	2.72512	1.21771
C	2.51941	3.30311	1.15375
C	3.49932	3.01011	0.28257
C	3.45871	2.04615	-0.87476
C	4.55476	0.95762	-0.83673
C	4.48633	0.05118	0.40406
C	5.25109	-1.28175	0.26667
C	4.64486	-2.327	-0.6921
C	3.43114	-3.12575	-0.14784
C	-2.36691	0.04716	-0.23499
C	-3.32528	0.70702	-0.89446
C	-4.66244	0.1073	-1.2717
C	-5.82235	0.92007	-0.74033
C	-6.78394	0.52627	0.11024
C	-6.96997	-0.8244	0.75211
C	-8.33038	-1.45924	0.40914
H	1.94886	-2.54944	2.18222
H	-1.16292	0.87966	1.28303
H	-1.02563	2.02434	-1.48186
H	0.5408	3.60206	-0.73012
H	-0.71405	-0.32613	2.64812
H	0.55325	3.30425	1.9677
H	1.15587	1.70256	1.61267
H	2.75139	4.0315	1.93297
H	4.45289	3.52992	0.4161
H	2.47801	1.56483	-0.93486
H	3.58109	2.61575	-1.80885
H	4.45047	0.35539	-1.74838
H	5.54782	1.42645	-0.89152
H	4.88829	0.59617	1.26778
H	3.43773	-0.14681	0.6489
H	6.2733	-1.06137	-0.07223
H	5.35651	-1.73885	1.2608

Table S18. *Cont.*

Atom	X	Y	Z
H	4.36782	-1.86115	-1.64522
H	5.41631	-3.06924	-0.9306
H	3.69792	-3.61343	0.79633
H	3.19081	-3.91887	-0.86925
H	-2.55538	-0.98773	0.06308
H	-3.17457	1.74589	-1.19386
H	-4.73693	0.08714	-2.37137
H	-4.71028	-0.93241	-0.93311
H	-5.85763	1.9518	-1.09973
H	-7.54463	1.2652	0.37848
H	-6.16423	-1.51033	0.47059
H	-6.90804	-0.70479	1.84421
H	-9.15722	-0.79951	0.69742
H	-8.41524	-1.6501	-0.66653
H	-8.46218	-2.41153	0.93476

Table S19. Coordinates (Ångstroms) for conformer (*M*, *R*)-C6 of neurymenolide A (Energy: -1159.523829 Hartree, Solvent: CH_3OH, Boltzmann %: 2.708040888).

Atom	X	Y	Z
O	1.19727	-1.74627	0.09846
C	2.09011	-1.94353	1.10039
C	2.00497	-1.21053	2.23556
C	0.9868	-0.21292	2.34352
C	0.09909	0.05165	1.31079
C	0.20235	-0.75305	0.12354
O	-0.47334	-0.68438	-0.89937
C	-0.9505	1.15719	1.47142
C	-0.37354	2.58232	1.38988
C	0.27645	3.20122	0.3925
O	1.00714	0.44088	3.52343
C	0.65121	2.69218	-0.97826
C	2.05939	3.08942	-1.37172
C	2.9545	2.36584	-2.06276
C	2.7844	0.97425	-2.61505
C	3.93395	0.00712	-2.26119
C	4.17675	-0.14899	-0.74846
C	4.88022	-1.46619	-0.36302
C	3.99892	-2.73007	-0.4421
C	3.10747	-2.9987	0.79627
C	-2.20126	1.04878	0.61625
C	-3.42627	0.90277	1.13615
C	-4.71164	0.89925	0.34215
C	-5.58853	-0.29305	0.65559
C	-6.26468	-1.06871	-0.20675
C	-6.32203	-0.96484	-1.70927
C	-6.01955	-2.30238	-2.40722
H	2.70516	-1.35767	3.05269
H	-1.32022	1.05852	2.50455
H	-0.58045	3.18288	2.2784
H	0.55316	4.24382	0.57303
H	0.24356	1.03469	3.67404
H	0.49033	1.61712	-1.06785
H	-0.04505	3.16068	-1.69693
H	2.34898	4.09955	-1.07049
H	3.92089	2.83346	-2.27272
H	1.83754	0.53955	-2.2768
H	2.71736	1.03703	-3.71237
H	3.69379	-0.96763	-2.70524
H	4.86321	0.34097	-2.74434
H	4.77905	0.69572	-0.39112
H	3.22149	-0.06791	-0.2195
H	5.74743	-1.60275	-1.02394
H	5.28737	-1.38113	0.65427

Table S19. *Cont.*

Atom	X	Y	Z
H	3.3687	-2.7003	-1.33897
H	4.64184	-3.61175	-0.55187
H	3.73543	-3.12165	1.6856
H	2.57695	-3.9486	0.6402
H	-2.07774	1.15605	-0.45832
H	-3.5359	0.79268	2.21971
H	-5.27504	1.81254	0.60136
H	-4.49071	0.96763	-0.72803
H	-5.68459	-0.52032	1.72057
H	-6.86393	-1.87983	0.21702
H	-7.33286	-0.64101	-2.00127
H	-5.63656	-0.1934	-2.07639
H	-5.00061	-2.63995	-2.18696
H	-6.71018	-3.08584	-2.07349
H	-6.11849	-2.20916	-3.49432

Table S20. Coordinates (Ångstroms) for conformer (*P*, *R*)-C1 of neurymenolide A (Energy: -1159.526518 Hartree, Solvent: CH_3OH, Boltzmann %: 49.4731114).

Atom	X	Y	Z
C	1.02138	-1.76542	-0.89595
C	1.76462	-2.21885	0.14292
C	0.70058	-0.65293	1.65868
C	-0.01268	-0.07583	0.55324
C	0.12395	-0.67205	-0.68905
O	-0.53067	-0.28662	-1.80296
C	-0.85141	1.16487	0.83328
C	-0.53735	2.32659	-0.11624
C	0.56906	3.08635	-0.09998
O	0.6373	-0.31462	2.84049
C	1.73786	2.96384	0.85687
C	3.09	3.37064	0.28768
C	3.76517	2.76431	-0.70212
C	3.32377	1.5743	-1.51247
C	4.2656	0.35198	-1.44434
C	4.38313	-0.26284	-0.0394
C	4.97013	-1.68834	-0.00558
C	4.13899	-2.80726	-0.66601
C	2.85174	-3.25216	0.07799
C	-2.35001	0.90904	0.89583
C	-2.96907	-0.27566	0.83798
C	-4.46207	-0.46857	0.97485
C	-5.05533	-1.23438	-0.18683
C	-6.10727	-0.89365	-0.94856
C	-6.97745	0.33285	-0.84958
C	-8.45888	-0.01499	-0.61236
H	-0.56043	1.48786	1.8417
H	-1.30802	2.56947	-0.85289
H	0.63904	3.89613	-0.82927
H	1.53348	3.60594	1.72591
H	1.7926	1.94407	1.25248
H	3.55522	4.23994	0.75571
H	4.7408	3.17769	-0.97382
H	2.31707	1.26515	-1.21307
H	3.25032	1.88562	-2.56569
H	3.87967	-0.39478	-2.14902
H	5.26504	0.62914	-1.80922
H	5.01395	0.38412	0.58369
H	3.3993	-0.25428	0.44091
H	5.95227	-1.66936	-0.49908
H	5.16301	-1.96787	1.03972
H	3.87851	-2.53671	-1.69655
H	4.77169	-3.70069	-0.73967
H	3.0988	-3.55641	1.10109

Table S20. *Cont.*

Atom	X	Y	Z
H	2.45128	-4.13434	-0.43663
H	-2.94863	1.81337	1.03844
H	-2.38447	-1.18788	0.70144
H	-4.64701	-1.04781	1.89527
H	-4.95166	0.50056	1.11165
H	-4.55434	-2.17966	-0.41158
H	-6.39248	-1.58989	-1.74257
H	-6.6284	1.00274	-0.05684
H	-6.89967	0.89713	-1.79098
H	-8.84241	-0.66789	-1.40519
H	-8.59245	-0.53572	0.34246
H	-9.07605	0.89016	-0.59532
H	-1.13798	0.46514	-1.66785
O	1.5855	-1.70073	1.3863
H	1.1382	-2.18632	-1.89108

Table S21. Coordinates (Ångstroms) for conformer (*P*, *R*)-C2 of neurymenolide A (Energy: -1159.524501 Hartree, Solvent: CH_3OH, Boltzmann %: 5.837827141).

Atom	X	Y	Z
C	0.60183	-2.04828	-0.51915
C	1.53547	-2.3063	0.43099
C	0.81251	-0.39985	1.75098
C	-0.0887	-0.03172	0.69844
C	-0.23418	-0.90156	-0.36804
O	-1.13366	-0.71287	-1.35682
C	-0.84134	1.29708	0.8057
C	-0.45783	2.2488	-0.32302
C	0.64714	3.00609	-0.39731
O	0.99091	0.1824	2.82075
C	1.75822	3.13301	0.61998
C	3.16374	3.26987	0.04784
C	3.7637	2.52192	-0.89323
C	3.20789	1.34367	-1.65089
C	4.08569	0.07417	-1.57457
C	4.26006	-0.46269	-0.1441
C	4.76057	-1.91751	-0.04633
C	3.81023	-3.02809	-0.53827
C	2.59368	-3.36706	0.36806
C	-2.33784	1.04325	0.93391
C	-3.33879	1.65877	0.28808
C	-4.80204	1.35495	0.52013
C	-5.51345	0.94452	-0.75004
C	-6.22012	-0.17616	-0.96798
C	-6.48575	-1.31477	-0.01704
C	-6.00894	-2.66887	-0.5733
H	-0.51998	1.7328	1.76019
H	-1.14093	2.29911	-1.17173
H	0.76695	3.62584	-1.28868
H	1.56449	4.03716	1.21601
H	1.72524	2.30258	1.33512
H	3.75373	4.07857	0.48324
H	4.79247	2.7891	-1.15362
H	2.2008	1.10319	-1.29652
H	3.10637	1.62325	-2.71093
H	3.61908	-0.68798	-2.21142
H	5.07311	0.27716	-2.01362
H	4.9699	0.18009	0.39228
H	3.31446	-0.35723	0.39475
H	5.69425	-2.00118	-0.62071
H	5.02992	-2.12953	0.99791
H	3.44735	-2.80783	-1.55
H	4.39059	-3.95562	-0.62135
H	2.93705	-3.58021	1.38675

Table S21. *Cont.*

Atom	X	Y	Z
H	2.1299	-4.28091	-0.02134
H	-2.59028	0.27104	1.66605
H	-3.1301	2.4428	-0.44248
H	-4.90383	0.59558	1.30142
H	-5.28204	2.27048	0.90369
H	-5.42941	1.65952	-1.57247
H	-6.6653	-0.29869	-1.95964
H	-6.01936	-1.13401	0.9571
H	-7.56915	-1.37549	0.16579
H	-6.48341	-2.88889	-1.53686
H	-4.92426	-2.67013	-0.72756
H	-6.25548	-3.48408	0.11591
H	-1.76859	-0.01183	-1.1221
O	1.59544	-1.54336	1.55405
H	0.51452	-2.67335	-1.40414

Table S22. Coordinates (Ångstroms) for conformer (*P*, *R*)-C3 of neurymenolide A (Energy: -1159.524676 Hartree, Solvent: CH_3OH, Boltzmann %: 7.030129125).

Atom	X	Y	Z
C	0.66117	-2.09942	-0.55506
C	1.61618	-2.34945	0.37587
C	0.89247	-0.4608	1.72103
C	-0.03211	-0.09908	0.68715
C	-0.18698	-0.96501	-0.38121
O	-1.10729	-0.77873	-1.35164
C	-0.79129	1.22459	0.80677
C	-0.3908	2.19266	-0.30187
C	0.70606	2.96402	-0.34246
O	1.08178	0.11651	2.79153
C	1.78722	3.1005	0.70473
C	3.20709	3.25758	0.17528
C	3.83733	2.54696	-0.77513
C	3.31359	1.39452	-1.59359
C	4.20936	0.13596	-1.55172
C	4.35736	-0.46269	-0.14261
C	4.85283	-1.92203	-0.1008
C	3.89372	-3.00915	-0.62755
C	2.6918	-3.3897	0.28252
C	-2.28918	0.96361	0.90241
C	-3.27883	1.58284	0.24214
C	-4.7456	1.26906	0.4363
C	-5.42572	0.88378	-0.85875
C	-6.05372	-0.26807	-1.14448
C	-6.25615	-1.46935	-0.25755
C	-7.74513	-1.78928	-0.02833
H	-0.48811	1.64574	1.77346
H	-1.04956	2.23626	-1.16978
H	0.8421	3.58899	-1.22785
H	1.56538	4.00002	1.29824
H	1.74512	2.26749	1.41653
H	3.78251	4.05061	0.65677
H	4.87154	2.82919	-0.99498
H	2.30301	1.12538	-1.27153
H	3.23009	1.71685	-2.64307
H	3.772	-0.60455	-2.23364
H	5.20397	0.37415	-1.95547
H	5.05821	0.15415	0.43457
H	3.40244	-0.37842	0.38307
H	5.78172	-1.9883	-0.68515
H	5.12905	-2.17378	0.93273
H	3.51343	-2.74058	-1.62121
H	4.47169	-3.93167	-0.76475
H	3.04822	-3.6187	1.29332

Table S22. *Cont.*

Atom	X	Y	Z
H	2.24053	-4.3027	-0.12302
H	-2.55389	0.18282	1.62108
H	-3.05747	2.3764	-0.47422
H	-4.86026	0.49267	1.19865
H	-5.23825	2.1743	0.82712
H	-5.38468	1.64626	-1.64074
H	-6.49038	-0.3616	-2.14307
H	-5.78273	-2.34044	-0.73447
H	-5.75765	-1.3372	0.70842
H	-8.24537	-0.97261	0.50401
H	-8.26787	-1.93702	-0.98057
H	-7.86218	-2.70344	0.56415
H	-1.75004	-0.09265	-1.09081
O	1.68498	-1.5941	1.50344
H	0.56774	-2.71936	-1.44303

Table S23. Coordinates (Ångstroms) for conformer (*P*, *R*)-C4 of neurymenolide A (Energy: -1159.525184 Hartree, Solvent: CH_3OH, Boltzmann %: 12.03680799).

Atom	X	Y	Z
C	1.36031	-2.14542	-0.63024
C	2.39474	-2.19502	0.24649
C	1.2767	-0.64966	1.75161
C	0.23159	-0.4863	0.78397
C	0.2568	-1.28765	-0.34379
O	-0.72695	-1.28978	-1.26879
C	-0.85605	0.55883	1.04423
C	-0.82264	1.67759	0.00622
C	0.04038	2.70335	-0.04846
O	1.36932	-0.10623	2.8519
C	1.16384	3.0291	0.90897
C	2.4361	3.57113	0.26795
C	3.12796	3.0739	-0.771
C	2.82786	1.84613	-1.59142
C	4.01676	0.86879	-1.72466
C	4.51026	0.31548	-0.37693
C	5.37225	-0.95889	-0.46981
C	4.67668	-2.24937	-0.94772
C	3.69032	-2.92419	0.04529
C	-2.21166	-0.12242	1.1875
C	-3.38078	0.24052	0.63894
C	-4.687	-0.48956	0.86351
C	-5.23599	-1.08083	-0.41712
C	-6.32776	-0.6984	-1.09931
C	-7.3001	0.40343	-0.76711
C	-8.74468	-0.10936	-0.62075
H	-0.61836	0.98302	2.0283
H	-1.56235	1.62153	-0.79291
H	-0.07782	3.40817	-0.8745
H	0.79927	3.80149	1.60238
H	1.39911	2.16305	1.53871
H	2.83118	4.47503	0.7354
H	4.02705	3.61999	-1.07244
H	1.9698	1.31156	-1.173
H	2.53279	2.16204	-2.6041
H	3.69543	0.04661	-2.3766
H	4.8494	1.36397	-2.24476
H	5.0976	1.09022	0.13258
H	3.6496	0.13505	0.27335
H	6.21117	-0.76221	-1.1527
H	5.82378	-1.15276	0.51335
H	4.15913	-2.08001	-1.90018
H	5.4552	-2.99415	-1.1554
H	4.1731	-3.05565	1.02023

Table S23. *Cont.*

Atom	X	Y	Z
H	3.4551	-3.92259	-0.34208
H	-2.19666	-0.9989	1.84157
H	-3.44381	1.11497	-0.01094
H	-4.52818	-1.29372	1.59477
H	-5.41088	0.20667	1.3041
H	-4.64872	-1.9078	-0.82361
H	-6.56209	-1.25159	-2.01345
H	-7.00316	0.93232	0.145
H	-7.27536	1.14705	-1.57767
H	-9.07307	-0.6272	-1.52957
H	-8.82958	-0.81372	0.21425
H	-9.43738	0.71952	-0.43799
H	-1.50808	-0.80377	-0.94667
O	2.3287	-1.51294	1.41973
H	1.38295	-2.71391	-1.55639

Table S24. Coordinates (Ångstroms) for conformer (*P*, *R*)-C5 of neurymenolide A (Energy: -1159.525116 Hartree, Solvent: CH_3OH, Boltzmann %: 11.20071241).

Atom	X	Y	Z
C	1.43532	-2.13128	-0.77821
C	2.479	-2.18642	0.087
C	1.30034	-0.80113	1.69754
C	0.24215	-0.62163	0.7467
C	0.29516	-1.347	-0.4303
O	-0.69415	-1.33496	-1.34915
C	-0.88448	0.36136	1.07446
C	-0.90706	1.53456	0.09845
C	-0.0965	2.60353	0.10302
O	1.37423	-0.33063	2.8325
C	1.00582	2.92976	1.0844
C	2.24195	3.59323	0.49028
C	2.9599	3.22112	-0.58263
C	2.73454	2.04842	-1.50191
C	3.96549	1.12786	-1.66164
C	4.43367	0.49695	-0.3394
C	5.36826	-0.71971	-0.48924
C	4.7641	-2.00656	-1.0864
C	3.81086	-2.82546	-0.17174
C	-2.21112	-0.37859	1.1939
C	-3.40487	-0.01495	0.70197
C	-4.68293	-0.79535	0.91563
C	-5.29171	-1.27441	-0.38432
C	-6.49491	-0.96624	-0.89516
C	-7.5533	-0.06033	-0.32146
C	-7.9512	1.06726	-1.29165
H	-0.65205	0.74086	2.07787
H	-1.64384	1.48482	-0.70408
H	-0.24863	3.34678	-0.68275
H	0.59465	3.62493	1.83139
H	1.29602	2.03668	1.64999
H	2.58625	4.47753	1.02979
H	3.82702	3.83949	-0.8342
H	1.88355	1.45142	-1.15949
H	2.46471	2.43012	-2.49872
H	3.69959	0.34418	-2.38238
H	4.79507	1.69073	-2.11333
H	4.95618	1.25936	0.25251
H	3.55799	0.22279	0.25577
H	6.2186	-0.42748	-1.12206
H	5.79524	-0.96143	0.49425
H	4.24965	-1.7903	-2.03106
H	5.59113	-2.68126	-1.34102
H	4.29725	-3.02363	0.79012

Table S24. *Cont.*

Atom	X	Y	Z
H	3.63055	-3.79304	-0.65467
H	-2.15156	-1.29784	1.78358
H	-3.50975	0.90236	0.11989
H	-4.46967	-1.66156	1.55769
H	-5.39536	-0.16431	1.46087
H	-4.65846	-1.95483	-0.95882
H	-6.75844	-1.42189	-1.8541
H	-8.44835	-0.66137	-0.10125
H	-7.23014	0.37275	0.63108
H	-7.10093	1.72666	-1.4988
H	-8.29893	0.65988	-2.24822
H	-8.75953	1.67645	-0.87257
H	-1.49233	-0.90125	-0.99632
O	2.38817	-1.58989	1.30471
H	1.4776	-2.63379	-1.74116

Table S25. Coordinates (Ångstroms) for conformer (*P*, *R*)-C6 of neurymenolide A (Energy: -1159.525354 Hartree, Solvent: CH_3OH, Boltzmann %: 14.42141196).

Atom	X	Y	Z
C	1.6779	-2.13627	-0.85591
C	2.60854	-2.17598	0.13045
C	1.21283	-0.82559	1.58803
C	0.27184	-0.66544	0.51755
C	0.48461	-1.38051	-0.6477
O	-0.38175	-1.38622	-1.68298
C	-0.91408	0.28037	0.7193
C	-0.8614	1.46614	-0.24121
C	-0.08617	2.55645	-0.1395
O	1.13762	-0.35864	2.72428
C	0.89867	2.89977	0.95512
C	2.17976	3.58285	0.49173
C	3.01879	3.2078	-0.4882
C	2.91787	2.01132	-1.39911
C	4.18852	1.13324	-1.43293
C	4.55227	0.52801	-0.06687
C	5.52616	-0.66542	-0.11941
C	5.01417	-1.96775	-0.76792
C	3.97303	-2.79265	0.03777
C	-2.22509	-0.49502	0.67655
C	-3.37547	-0.12618	0.09478
C	-4.65185	-0.93652	0.14646
-C	-5.73355	-0.22496	0.93089
C	-6.89216	0.27395	0.46983
C	-7.42879	0.24751	-0.93786
C	-8.78347	-0.47746	-1.03949
H	-0.81252	0.65685	1.74563
H	-1.51134	1.40902	-1.11521
H	-0.17508	3.30541	-0.92956
H	0.3993	3.58845	1.65263
H	1.14118	2.01132	1.54978
H	2.4418	4.48553	1.04678
H	3.89496	3.84082	-0.65854
H	2.06169	1.38978	-1.11916
H	2.72262	2.36539	-2.42322
H	4.01606	0.33488	-2.16575
H	5.03731	1.71993	-1.8128
H	5.00065	1.30914	0.56039
H	3.63435	0.23667	0.45144
H	6.42693	-0.35493	-0.66802
H	5.8601	-0.89487	0.90228
H	4.61126	-1.7688	-1.76875
H	5.87595	-2.63056	-0.91613
H	4.34497	-2.97266	1.05277

Table S25. *Cont.*

Atom	X	Y	Z
H	3.86763	-3.76883	-0.45044
H	-2.1984	-1.44482	1.21809
H	-3.45002	0.8266	-0.43226
H	-4.9917	-1.14161	-0.87562
H	-4.44226	-1.90681	0.61722
H	-5.51504	-0.10255	1.99443
H	-7.54858	0.76013	1.19739
H	-7.56302	1.28472	-1.27968
H	-6.71255	-0.21325	-1.62656
H	-8.68632	-1.53335	-0.76341
H	-9.52445	-0.02416	-0.37064
H	-9.17834	-0.428	-2.06028
H	-1.22928	-0.97183	-1.43873
O	2.35858	-1.58528	1.32865
H	1.84892	-2.63241	-1.80775
